# Comparative and Evolutionary Aspects of Gonadotropin-Inhibitory Hormone and FMRFamide-Like Peptide Systems

**DOI:** 10.3389/fnins.2018.00747

**Published:** 2018-10-18

**Authors:** Takayoshi Ubuka, Kazuyoshi Tsutsui

**Affiliations:** Laboratory of Integrative Brain Sciences, Department of Biology and Center for Medical Life Science, Waseda University, Shinjuku, Japan

**Keywords:** gonadotropin-inhibitory hormone (GnIH), neuropeptide FF (NPFF), FMRFamide-like peptide (FLP), GPR147, GPR74, chordates, nematodes, *C. elegans*

## Abstract

Gonadotropin-inhibitory hormone (GnIH) is a hypothalamic neuropeptide that was found in the brain of Japanese quail when investigating the existence of RFamide peptides in birds. GnIH was named because it decreased gonadotropin release from cultured anterior pituitary, which was located in the hypothalamo-hypophysial system. GnIH and GnIH precursor gene related peptides have a characteristic C-terminal LPXRFamide (X = L or Q) motif that is conserved in jawed vertebrates. Orthologous peptides to GnIH are also named RFamide related peptide or LPXRFamide peptide from their structure. A G-protein coupled receptor GPR147 is the primary receptor for GnIH. Similarity-based clustering of neuropeptide precursors in metazoan species indicates that GnIH precursor of vertebrates is evolutionarily related to FMRFamide precursor of mollusk and nematode. FMRFamide peptide is the first RFamide peptide that was identified from the ganglia of the venus clam. In order to infer the evolutionary history of the GnIH-GnIH receptor system we investigate the structural similarities between GnIH and its receptor and well-studied nematode *Caenorhabditis elegans* (*C. elegans*) FMRFamide-like peptides (FLPs) and their receptors. We also compare the functions of FLPs of nematode with GnIH of chordates. A multiple sequence alignment and phylogenetic analyses of GnIH, neuropeptide FF (NPFF), a paralogous peptide of GnIH, and FLP precursors have shown that GnIH and NPFF precursors belong to different clades and some FLP precursors have structural similarities to either precursor. The peptide coding regions of FLP precursors in the same clade align well with those of GnIH or NPFF precursors. Alignment of GnIH (LPXRFa) peptides of chordates and FLPs of *C. elegans* grouped the peptides into five groups according to the last C-terminal amino acid sequences, which were MRFa, LRFa, VRFa, IRFa, and PQRFa. Phylogenetic analysis of receptors suggested that GPR147 has evolutionary relationships with FLP receptors, which regulate reproduction, aggression, locomotion, and feeding. GnIH and some FLPs mediate the effect of stress on reproduction and behavior, which may also be a conserved property of these peptide systems. Future studies are needed to investigate the mechanism of how neuropeptide precursor genes are mutated to evolve new neuropeptides and their inheritance.

## Introduction

A hypothalamic neuropeptide gonadotropin-inhibitory hormone (GnIH) was discovered in the Japanese quail (*Coturnix japonica*) brain, while studying the existence of Arg-Phe-NH_2_ (RFamide) peptides in birds, which have a characteristic C-terminal RFamide sequence (Tsutsui et al., 2000). Phe-Met-Arg-Phe-NH_2_ (FMRFamide) is the first RFamide peptide that was identified in the venus clam *Macrocallista nimbosa* ganglia, which has a cardioexcitatory function (Price and Greenberg, 1977). Since then, multiple RFamide peptides acting as hormones, neuromodulators and neurotransmitters have been found in cnidarians, nematodes, annelids, mollusks, and arthropods. Multiple immunohistochemical studies using antibodies against RFamide peptides of invertebrates suggested the presence of RFamide peptides in the central nervous system of vertebrates.

Tsutsui et al. (2000) have successfully isolated a peptide from 500 Japanese quail brains using high-performance liquid chromatography combined with a competitive enzyme-linked immunosorbent assay with an antibody against Arg-Phe-NH_2_. The C-terminal structure of the isolated peptide SIKPSAYLPLRFamide (Table 1) was found to be identical to the chicken LPLRFamide that was reported as the first RFamide peptide isolated in vertebrates (Dockray et al., 1983). However, the previously reported chicken LPLRFamide peptide can be the fragment of the chicken GnIH peptide that was identified to have a sequence of SIRPSAYLPLRFamide in a recent study (McConn et al., 2014). GnIH was named “gonadotropin-inhibitory hormone” because it decreased gonadotropin release from cultured quail anterior pituitary gland and located in the hypothalamo-hypophysial system (Tsutsui et al., 2000; for reviews see, Tsutsui et al., 2015, 2017; Ubuka et al., 2016).

**Table 1:** Representative GnIH (LPXRFa) peptides of chordates.

**Animal**	**Peptide sequences**	**References**
Human	**MPHSFANLPLRFa** (RFRP-1), SAGATANLPLRSa (RFRP-2), **VPNLPQRFa** (RFRP-3)	Ubuka et al., 2009c
Quail	VPNSVANLPLRFa (GnIH-RP-1), **SIKPSAYLPLRFa** (GnIH), **SSIQSLLNLPQRFa** (GnIH-RP-2)	Tsutsui et al., 2000; Satake et al., 2001
Newt	**MPHASANLPLRFa** (nLPXRFa-2), **SVPNLPQRFa** (nLPXRFa-1), **SIQPLANLPQRFa** (nLPXRFa-3), **APSAGQFIQTLANLPQRFa** (nLPXRFa-4)	Chowdhury et al., 2011
Coelacanth	FSNSVINLPLRFa (LPXRFa-1), LSQSLANLPLRLa (LPXRFa-2), IPMAIPNLPQRFa (LPXRFa-3), SFMQPLANLPQRFa (LPXRFa-4), FIQSVANLPQRFa (LPXRFa-5)	Muñoz-Cueto et al., 2017
Zebrafish	PAHLHANLPLRFa (LPXRFa-1), STINLPQRFa (LPXRFa-2), SGTGPSATLPQRFa (LPXRFa-3)	Muñoz-Cueto et al., 2017
Gar	LYHSVTNLPLRFa (LPXRFa-1), ASQPVANLPLRFa (LPXRFa-2), AALNLPQRFa (LPXRFa-3)	Muñoz-Cueto et al., 2017
Lamprey	**SGVGQGRSSKTLFQPQRFa** (lLPXRFa-1a), **SEPFWHRTRPQRFa** (lLPXRFa-2)	Osugi et al., 2012
Amphioxus	**WDEAWRPQRFa** (PQRFa-1), **GDHTKDGWRPQRFa** (PQRFa-2), **GRDQGWRPQRFa** (PQRFa-3)	Osugi et al., 2014

*Biochemically identified mature peptides are shown in bold (Ubuka et al., 2016)*.

## Structure of GNIH peptides and their positions in their precursor proteins

**Figure 1:**
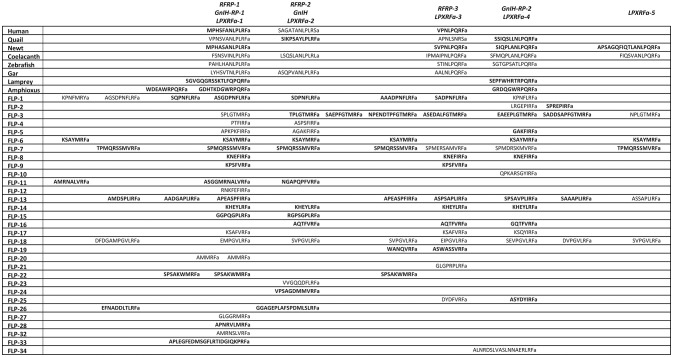
A schematic representation of the multiple sequence alignment of human, quail, newt, coelacanth, zebrafish, gar, lamprey, amphioxus GnIH and *C. elegans* FMRFamide-like peptide (FLP) precursors highlighting the sequences of identified and predicted biologically active peptides. Human, quail, newt, coelacanth, zebrafish, gar, lamprey, amphioxus GnIH and *C. elegans* FLP precursor polypeptides were aligned by EMBL-EBI Clustal Omega Multiple Sequence Alignment software. The positions of the identified or predicted endogenous peptide sequences in their precursors are shown in bold or normal font, respectively. The full alignment of the precursors are shown in Supplementary Figure 1. Accession numbers are human (*Homo sapiens*) GnIH precursor (NP_071433.3), Japanese quail (*Coturnix japonica*) GnIH precursor (XP_015709159.1), Japanese fire belly newt (*Cynops pyrrhogaster*) GnIH precursor (BAJ78290.1), West Indian Ocean coelacanth (*Latimeria chalumnae*) GnIH precursor (XP_005993154.1), zebrafish (*Danio rerio*) GnIH precursor (NP_001076418.1), spotted gar (*Lepisosteus oculatus*) GnIH precursor (XP_015213317.1), sea lamprey (*Petromyzon marinus*) GnIH precursor (BAL52329.1), Japanese amphioxus (*Branchiostoma japonicum*) GnIH precursor (BAO77760.1), *C. elegans* (*Caenorhabditis elegans*) FLP-1 precursor (AAC46464.1), FLP-2 precursor (NP_001024945.1), FLP-3 precursor (AAC08940.1), FLP-4 precursor (AAC08941.1), FLP-5 precursor (AAC08942.1), FLP-6 precursor (AAC08943.1), FLP-7 precursor (AAC08944.1), FLP-8 precursor (AAC08945.1), FLP-9 precursor (AAC08946.1), FLP-10 precursor (AAC08947.1), FLP-11 precursor (NP_001024752.1), FLP-12 precursor (AAC08950.1), FLP-13 precursor (AAC08951.1), FLP-14 precursor (NP_499682.2), FLP-15 precursor (NP_499820.1), FLP-16 precursor (NP_001022091.1), FLP-17 precursor (NP_503051.1), FLP-18 precursor (NP_508514.2), FLP-19 precursor (NP_509776.1), FLP-20 precursor (NP_509574.2), FLP-21 precursor (NP_505011.2), FLP-22 precursor (NP_492344.2), FLP-23 precursor (AAY18633.1), FLP-24 precursor (AAW78866.1), FLP-25 precursor (NP_001022665.1), FLP-26 precursor (NP_741827.1), FLP-27 precursor (NP_495111.1), FLP-28 precursor (NP_001024947.1), FLP-32 precursor (NP_510551.2), FLP-33 precursor (NP_871818.1), FLP-34 precursor isoform 1 (FLP-34; NP_001300170.1), FLP-34 precursor isoform 2 (FLP-34'; NP_503365.1).

In the following year of the discovery of quail GnIH, the precursor cDNA of quail GnIH was cloned and sequenced (Satake et al., 2001). GnIH precursor is composed of 173 amino acid residues encompassing GnIH as well as two GnIH-related peptides (GnIH-RP-1 and GnIH-RP-2; Figure 1, Supplementary Figure 1, Table 1). GnIH and GnIH-related peptide sequences are flanked by an amidation signal glycine at the C-terminal as well as basic amino acids (arginine or lysine) as endoproteolytic sites on each end (Supplementary Figure 1). The translated and processed GnIH and GnIH-RPs all possess a C-terminal LPXRFamide (X = L or Q) sequence (Figure 1, Supplementary Figure 1, Table 1). Mass spectrometry has also identified the mature peptide structure of GnIH-RP-2 in addition to GnIH (Satake et al., 2001). Within the class of birds, mature GnIH peptides were also isolated in European starlings (Ubuka et al., 2008), zebra finch (Tobari et al., 2010), and chicken (McConn et al., 2014; Ubuka et al., 2016). A cDNA encoding LPXRFamide peptides in the brain of red-eared slider was recently cloned and immunoaffinity purification and mass spectrometry identified three mature LPXRFamide peptides that were encoded in the precursor protein (Ukena et al., 2016).

In mammals, cDNAs encoding C-terminal LPXRFamide peptides were found in the gene database (Hinuma et al., 2000). Mammalian LPXRFamide peptides and related peptides were named RFamide related peptides (RFRPs) from their C-terminal structure (Hinuma et al., 2000). Human LPXRFamide precursor cDNA encodes three RFRPs (RFRP-1, RFRP-2, and RFRP-3). However, although RFRP-1 and RFRP-3 possess a C-terminal LPXRFamide (X = L or Q) motif, the predicted C-terminal sequence of RFRP-2 is RSamide (Hinuma et al., 2000; Ubuka et al., 2009c; Figure 1, Supplementary Figure 1, Table 1). The LPLRF, LPLRS, and LPQRF sequences are followed by glycine as an amidation signal, followed by arginine or lysine at the C-terminus in the human LPXRFamide peptide precursor as in birds (Supplementary Figure 1). When human GnIH (RFRP) precursor protein was aligned with GnIH precursor protein of quail, human RFRP-1, and human RFRP-2 align with quail GnIH-RP-1 and quail GnIH, respectively (Figure 1, Supplementary Figure 1). Human RFRP-3 aligns with an LPXRFa-like peptide that has a C-terminal LSNRSamide sequence but not with quail GnIH-RP-2 (Figure 1, Supplementary Figure 1). In mammals, bovine RFRP-1 (Fukusumi et al., 2001) and -3 (Yoshida et al., 2003), rat RFRP-3 (Ukena et al., 2002), Siberian hamster RFRP-1 and -3 (Ubuka et al., 2012a), macaque RFRP-3 (Ubuka et al., 2009b), and human RFRP-1 and -3 (Ubuka et al., 2009c) are identified as mature peptides by biochemical experiments (Ubuka et al., 2016).

In amphibians, bullfrog LPXRFamide peptide was identified and named as frog growth hormone-releasing hormone (fGRP) as the peptide stimulated growth hormone release (Koda et al., 2002). The precursor cDNA encoded four LPXRFamide peptides that were named as fGRP, fGRP-RP-1, fGRP-RP-2, and fGRP-RP-3 (Sawada et al., 2002a) and all peptides were identified as mature peptides (Ukena et al., 2003a). In the same year fGRP was independently identified in European green frog and named as Rana RFamide (R-RFa) Chartrel et al., 2002). LPXRFamide peptide precursor cDNA was also cloned from Japanese red-bellied newt (Chowdhury et al., 2011). The deduced precursor protein encompasses four LPXRFamide peptides that were named as nLPXRFa-1,-2,-3,-4, which were all identified as endogenous mature peptides (Chowdhury et al., 2011; Figure 1, Supplementary Figure 1, Table 1). Newt LPXRFa-2, LPXRFa-1, and LPXRFa-3 align with human RFRP-1/quail GnIH-RP-1, human RFRP-3, and quail GnIH-RP-2, respectively (Figure 1, Supplementary Figure 1, Table 1). Coelacanth is a lobe-finned fish that is evolutionarily related to tetrapods. Coelacanth LPXRFamide precursor encompasses four LPXRFamide peptides and one LPXRFamide-like peptide (Figure 1, Supplementary Figure 1, Table 1). Coelacanth LPXRFa-1, LPXRFa-2, LPXRFa-3, LPXRFa-4 align to human RFRP-1/quail GnIH-RP-1, human RFRP-2/quail GnIH, human RFRP-3, and quail GnIH-RP-2, respectively (Figure 1, Supplementary Figure 1, Table 1). Coelacanth LPXRFa-5 aligns with newt nLPXRFa-4 (Figure 1, Supplementary Figure 1, Table 1).

In teleost fishes, a cDNA encoding three LPXRFamide peptides [goldfish (gf) LPXRFa-1,-2, and -3] was first cloned from goldfish and gf LPXRFa-3 was identified as a mature peptide (Sawada et al., 2002b). The zebrafish LPXRFamide precursor gene also encodes three LPXRFamide peptides (Figure 1, Supplementary Figure 1, Table 1). Zebrafish LPXRFa-1, LPXRFa-2, and LPXRFa-3 align with human RFRP-1/quail GnIH-RP-1, human RFRP-3, and quail GnIH-RP-2, respectively (Figure 1, Supplementary Figure 1, Table 1). Spotted gar a ray-finned fish that diverged from teleost fishes before teleost specific genome duplication is regarded as an ideal model to study the evolution of fish (Braasch et al., 2016; Muñoz-Cueto et al., 2017). Three LPXRFamide peptides are found in the gar LPXRFa precursor polypeptide. Gar LPXRFa-1, LPXRFa-2, and LPXRFa-3 align to human RFRP-1/quail GnIH-RP-1, human RFRP-2/quail GnIH, and human RFRP-3, respectively (Figure 1, Supplementary Figure 1, Table 1). Lamprey is a jawless fish (agnathan) that is regarded as one of the most basal vertebrates. The lamprey precursor protein possesses C-terminal QPQRFamide and RPQRFamide peptides, but not an LPXRFamide (X = L or Q) peptide. Lamprey LPXRFa-1a and LPXRFa-2 align with human RFRP-1/quail GnIH-RP-1 and quail GnIH-RP-2, respectively (Osugi et al., 2012; Figure 1, Supplementary Figure 1, Table 1). LPXRFamide peptide precursor gene was further searched in amphioxus, one of the most basal chordates (protochordates; Osugi et al., 2014). The C-termini of the amphioxus LPXRFamide-like peptides were RPQRFamide, and again LPXRFamide peptide (X = L or Q) was not found. Three of the RPQRFamide peptides were identified as mature peptides (Osugi et al., 2014; Table 1). Amphioxus PQRFa-2 and PQRFa-3 align with human RFRP-1/quail GnIH-RP-1 and quail GnIH-RP-2, respectively (Figure 1, Supplementary Figure 1, Table 1). These results suggest that the C-terminal LPXRFamide (X = L or Q) motif was evolved in gnathostomes (jawed vertebrates; Table 1).

**Figure 2:**
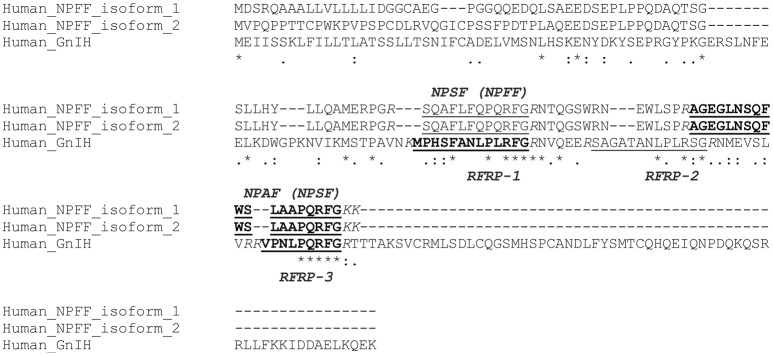
A multiple sequence alignment of human GnIH and NPFF precursors. Human GnIH and NPFF precursor polypeptides were aligned by CLUSTAL W Multiple Sequence Alignment. Multiple alignment parameters were as follows: Gap open penalty 10, Gap extension penalty 0.05, Hydrophilic residues GPSNDQERK, Weight matrix GONNET. Identified or predicted endogenous peptide sequences are underlined. Biochemically identified endogenous peptide sequences are shown in bold. Glycine (G) is an amidation signal. Lysine (K) and/or arginine (R) as endoproteolytic basic amino acids are Italicized. Accession numbers are human (*Homo sapiens*) GnIH precursor [Human_GnIH (RFRP), NP_071433.3], human NPFF precursor isoform 1 (Human_NPFF_isoform_1, NP_003708.1), human NPFF precursor isoform 2 (Human_NPFF_isoform_2, NP_001307225.1). Asterisk (^*^) indicates positions which have a single, fully conserved residue. Colon (:) indicates conservation between groups of strongly similar properties – scoring >0.5 in the Gonnet PAM 250 matrix. Period (.) indicates conservation between groups of weakly similar properties—scoring 60.5 in the Gonnet PAM 250 matrix.

Neuropeptide FF (NPFF, FLFQPQRFamide) was originally isolated from the bovine brain using the antibody against FMRFamide (Yang et al., 1985). The antibody also isolated a related peptide (AGEGLSSPFWSLAAPQRFamide) from the bovine brain, which was named neuropeptide AF (NPAF; Yang et al., 1985). Both NPFF and NPAF were shown to modulate pain sensitivity in rats (Yang et al., 1985). The NPFF precursor gene was identified in the human gene (Perry et al., 1997). There are two isoforms in the human NPFF precursor protein but both proteins encode the same two C-terminal PQRFamide peptides (Figure 2). The peptides having the sequences of SQAFLFQPQRFamide (named NPSF in Perry et al., 1997) and AGEGLNSQFWSLAAPQRFamide (named NPAF in Perry et al., 1997 and Burlet-Schiltz et al., 2002) were predicted (Perry et al., 1997; Figure 2). However, only NPAF and the partial NPAF which has a sequence of SLAAPQRFamide (named NPSF in Burlet-Schiltz et al., 2002) were isolated in the human cerebrospinal fluid and their structure was determined by mass spectrometry (Burlet-Schiltz et al., 2002; Figure 2). SQAFLFQPQRFamide (named NPSF in Perry et al., 1997) or its partial FLFQPQRFamide (the same sequence as bovine NPFF) was not isolated in the human cerebrospinal fluid (Burlet-Schiltz et al., 2002; Figure 2). When human NPFF precursor isoforms were aligned with human GnIH precursor, NPSF (Perry et al., 1997) that includes bovine NPFF sequence aligns with human RFRP-1 (Ubuka et al., 2009c; Figure 2). NPAF (Perry et al., 1997; Burlet-Schiltz et al., 2002) aligns with human RFRP-3 (Ubuka et al., 2009c; Figure 2).

## GNIH receptor and cell signaling

Hinuma et al. (2000) identified a specific receptor for GnIH (RFRP) and named it OT7T022, which is identical to GPR147. Bonini et al. (2000) reported two G-protein coupled receptor (GPCR) for NPFF, and named them as NPFF1 which is identical to GPR147 and NPFF2 which is identical to GPR74. GPR147 and GPR74 are paralogous (Fredriksson et al., 2003). The binding affinities and their signal transduction pathways were tested for GPR147 and GPR74, using GnIHs (RFRPs) and NPFF. GnIHs (RFRPs) had a higher affinity for GPR147, whereas NPFF had a potent agonistic activity for GPR74 (Bonini et al., 2000; Liu et al., 2001; Yoshida et al., 2003; Ikemoto and Park, 2005; Yin et al., 2005), suggesting that GPR147 (NPFF1, OT7T022) is the primary receptor for GnIH.

GnIH (RFRPs) suppresses cAMP production in cells transfected with chicken and rat GPR147 suggesting that GPR147 couples to G_α*i*_ protein which inhibits adenylate cyclase (AC) (Hinuma et al., 2000; Shimizu and Bédécarrats, 2010). The GnIH cell signaling pathway has been precisely investigated in LβT2 cells, a mouse gonadotrope cell line (Son et al., 2012). Mouse GnIHs (RFRPs) suppressed gonadotropin-releasing hormone (GnRH)-induced cAMP signaling, extracellular signal-regulated kinase (ERK) phosphorylation as well as gonadotropin subunit gene transcription by inhibiting the protein kinase A (PKA) pathway (Son et al., 2012; Ubuka et al., 2013). Because GnIH neurons extend their axons to GnRH neurons and GnRH neurons express GPR147 in birds and mammals (Ubuka et al., 2008, 2012a), GnIH cell signaling pathway was further investigated in GT1-7, a mouse GnRH neuronal cell line (Son et al., 2016). It was found that GnIH suppresses the effect of vasoactive intestinal polypeptide on AC activity, p38 and ERK phosphorylation, and c-Fos mRNA expression in GT1-7 cells (Son et al., 2016). These results suggest that GnIH specifically inhibits the AC/cAMP/PKA pathway in gonadotropes and GnRH neurons at least in birds and mammals (Son et al., 2012, 2016).

GnIH (RFRP-3) rapidly and repeatedly inhibits the firing of GnRH neurons as well, which was shown in the adult mice (Ducret et al., 2009). It was further shown that GnIH (RFRP-3) produces a non-desensitizing hyperpolarization in vesicular glutamate transporter 2 (vGluT2)-GnRH neurons by a direct postsynaptic Ba^2+^-sensitive K^+^ current mechanism (Wu et al., 2009).

## Regulation of physiology and behavior by GNIH in chordates

### Location of GnIH neurons and GnIH receptors in the brain

GnIH neuronal cell bodies are clustered in the paraventricular nucleus (PVN) of the hypothalamus in quail (Ubuka et al., 2003; Ukena et al., 2003b), house and song sparrows (Bentley et al., 2003), white-crowned sparrows (Osugi et al., 2004; Ubuka et al., 2012b), European starlings (Ubuka et al., 2008), and zebra finch (Tobari et al., 2010). GnIH-immunoreactive (ir) neuronal fibers are distributed in the diencephalic and mesencephalic areas in the brain in quail (Ukena et al., 2003b), European starlings (Ubuka et al., 2008), zebra finch (Tobari et al., 2010), and white-crowned sparrows (Ubuka et al., 2012b). GnIH neuronal axons terminate on GnRH-1 neurons in the preoptic area (POA), which release GnRH-1 at the median eminence and stimulate gonadotropin secretion from the anterior pituitary gland (King and Millar, 1982; Miyamoto et al., 1982; Sharp et al., 1990; Ubuka and Bentley, 2009, 2010; Ubuka et al., 2009a). GnIH neuronal axons also terminate on GnRH-2 neurons in the midbrain of European starlings and house sparrows (Bentley et al., 2003; Ubuka et al., 2008; Ubuka and Bentley, 2010), which stimulates reproductive behavior (Miyamoto et al., 1984; Maney et al., 1997; Temple et al., 2003; Barnett et al., 2006). It was clearly shown that GPR147 mRNA is expressed in GnRH-1 and GnRH-2 neuronal cell bodies in European starling (Ubuka et al., 2008).

GnIH neuronal cell bodies are clustered in the medial hypothalamic area, but in different brain regions or nuclei in mammals. In hamsters and mice, a cluster of GnIH neuronal cell bodies exists in the dorsomedial hypothalamic area (DMH) (Kriegsfeld et al., 2006; Ubuka et al., 2012a), whereas in rats it is in the periventricular nucleus (PerVN), and the portion between the dorsomedial (DMN) and the ventromedial (VMN) nuclei of the hypothalamus (Hinuma et al., 2000; Legagneux et al., 2009). In the macaque brain, a cluster of GnIH neuronal cell bodies principally exists in the intermediate periventricular nucleus (IPe) of the hypothalamus (Ubuka et al., 2009b), whereas in sheep it exists in the DMN and PVN (Clarke et al., 2008). GnIH-ir neuronal fibers are also widely located in the diencephalic, mesencephalic and limbic brain regions in mammals (Yano et al., 2003; Johnson et al., 2007; Ubuka et al., 2009b, 2012a). Studies in macaque, sheep and mice showed that GnIH-ir fibers are in close proximity to GnRH-1, dopamine, proopiomelanocortin (POMC), GnRH-2, neuropeptide Y (NPY), orexin, melanin-concentrating hormone (MCH), corticotrophin-releasing hormone (CRH), oxytocin, and kisspeptin neurons (Qi et al., 2009; Ubuka et al., 2009b; Poling et al., 2013). Five to ten percentage of kisspeptin neurons in the anteroventral periventricular (AVPV) region express GPR147 or GPR74, whereas approximately 25% express GPR147 or GPR74 in kisspeptin neurons in the arcuate nucleus in mice (Poling et al., 2013).

GnIH neuronal cell bodies are located in the nucleus accumbens, PVN, and upper medulla, and fibers contact the lateral processes of serotonin-ir neurons in the paraventricular organ (PVO) in the Japanese grass lizard (Kawano et al., 2006). On the other hand, GnIH neuronal cell bodies are restricted to the periventricular hypothalamic nucleus, and the fibers are densely distributed in the median eminence of red-eared slider turtle (Ukena et al., 2016).

GnIH neuronal cell bodies constitute two subpopulations in the telencephalon and diencephalon and the highest number of cell bodies are located in the POA and suprachiasmatic areas of the anuran amphibian *Rana esculenta* (Pinelli et al., 2015). GnIH neuronal cell bodies also exist in the medial septum, anterior commissure, dorsal hypothalamus, PerVN of the hypothalamus, and posterior tuberculum. GnIH neuronal fibers are only occasionally present in the median eminence. GnIH neuronal fibers exist in close proximity to GnRH cell bodies (Pinelli et al., 2015). GnIH (LPXRFa) neuronal cell bodies are located in the nucleus posterioris periventricularis and the nervus terminalis, and the fibers extend to nucleus lateralis tuberis pars posterioris and pituitary in goldfish and sockeye salmon (Sawada et al., 2002b; Amano et al., 2006). Sea bass GnIH (sbLPXRFa) neuronal cell bodies exist in the olfactory bulbs-terminal nerve, ventral telencephalon, caudal POA, dorsal mesencephalic tegmentum and rostral rhombencephalon, and fibers are widely distributed including the pituitary. GnIH (sbLPXRFa) neuronal fibers exist close to luteinizing hormone (LH), follicle-stimulating hormone (FSH), and growth hormone (GH) cells (Paullada-Salmerón et al., 2016a).

### Regulation of reproductive activity

Abundant GnIH neuronal fibers exist at the median eminence in quail (Tsutsui et al., 2000; Ubuka et al., 2003; Ukena et al., 2003b) as well as in other birds (Bentley et al., 2003; Osugi et al., 2004), suggesting direct action of GnIH on gonadotropin secretion from the pituitary in birds (Tsutsui et al., 2000; Ciccone et al., 2004; Osugi et al., 2004; Ubuka et al., 2006). GPR147-positive cells are co-localized with LHβ or FSHβ mRNA containing cells the chicken pituitary (Maddineni S. et al., 2008). Abundant GnIH neuronal fibers also exist in the median eminence of sheep (Clarke et al., 2008), macaque (Ubuka et al., 2009b), and humans (Ubuka et al., 2009c), and GPR147 mRNA is expressed in the gonadotropes in the human pituitary (Ubuka et al., 2009c), suggesting that GnIH also directly inhibits gonadotropin secretion in mammals (Clarke et al., 2008; Murakami et al., 2008; Kadokawa et al., 2009; Sari et al., 2009; Pineda et al., 2010a; Smith et al., 2012). However, GnIH may not directly inhibit gonadotropin secretion in some birds and rodents, because there are only few or no GnIH neuronal fibers in the median eminence in Rufous-winged sparrows (Small et al., 2008), hamsters (Kriegsfeld et al., 2006; Ubuka et al., 2012a), and rats (Rizwan et al., 2009).

GnIH neuronal axon terminals are found in close contact with GnRH neurons in birds (Bentley et al., 2003; Ubuka et al., 2008; Tobari et al., 2010), rodents (Kriegsfeld et al., 2006; Ubuka et al., 2012a), monkeys (Ubuka et al., 2009b), and humans (Ubuka et al., 2009c), and GnIH receptor mRNA or protein is expressed in GnRH neurons in birds (Ubuka et al., 2008) and mammals (Ubuka et al., 2012a). Therefore, in addition to directly regulating the pituitary, GnIH may inhibit gonadotropins secretion by decreasing the activity of GnRH neurons (Kriegsfeld et al., 2006; Johnson et al., 2007; Anderson et al., 2009; Ducret et al., 2009; Wu et al., 2009; Pineda et al., 2010a,b; Ubuka et al., 2012a; Fraley et al., 2013; Glanowska et al., 2014; Gojska et al., 2014; Xiang et al., 2015). GnIH may also act on kisspeptin neurons to modulate reproduction in mammals (Rizwan et al., 2012).

By controlling gonadotropin secretion, GnIH regulates reproductive development and maintenance in birds (Ubuka et al., 2003, 2006; Shimizu and Bédécarrats, 2006; Maddineni S. et al., 2008; Joseph et al., 2009) and mammals (Yano et al., 2004; Quennell et al., 2010; Sethi et al., 2010; Iwasa et al., 2012; Losa-Ward et al., 2012; Poling et al., 2012; Jørgensen et al., 2014; León et al., 2014; Soga et al., 2014; Zhao et al., 2014; Semaan and Kauffman, 2015; Xiang et al., 2015). GnIH regulates estrous or menstrual cycle in female mammals (Kriegsfeld et al., 2006; Gibson et al., 2008; Smith et al., 2010; Molnár et al., 2011; Clarke et al., 2012; Li et al., 2012; Salehi et al., 2013; Jørgensen et al., 2014; Russo et al., 2015). GnIH may also regulate seasonal reproduction in birds (Bentley et al., 2003; Ubuka et al., 2005; Small et al., 2008; Chowdhury et al., 2010; Surbhi et al., 2015) and mammals (Dardente et al., 2008; Revel et al., 2008; Smith et al., 2008; Gingerich et al., 2009; Mason et al., 2010; Ubuka et al., 2012a; Harbid et al., 2013; Henson et al., 2013; Janati et al., 2013; Klosen et al., 2013; Ikeno et al., 2014; Jafarzadeh Shirazi et al., 2014; Piekarski et al., 2014; Sáenz de Miera et al., 2014).

GnIH neuronal cell bodies exist in the posterior periventricular nucleus (NPPv) of teleost fishes (Ogawa and Parhar, 2014; Biswas et al., 2015; Di Yorio et al., 2016; Ogawa et al., 2016; Paullada-Salmerón et al., 2016a). They also exist in the ventral zone of the periventricular hypothalamus of zebrafish (Spicer et al., 2017), the periventricular preoptic nucleus (NPP) as well as magnocellular preoptic nucleus (NPOm) of Indian major carp (Biswas et al., 2015). In goldfish, Indian major carp, cichlid and sea bass, GnIH cells exist in the terminal nerve/olfactory bulbs (Biswas et al., 2015; Di Yorio et al., 2016; Paullada-Salmerón et al., 2016a). GnIH cells also exist in the dorsal mesencephalic tegmentum and rhombencephalon of sea bass as well as Indian major carp (Biswas et al., 2015; Paullada-Salmerón et al., 2016a). GnIH neuronal fibers innervate broad brain area in fishes, such as olfactory bulb, ventral and dorsal telencephalon, preoptic area, hypothalamus, and midbrain area (Ogawa and Parhar, 2014; Biswas et al., 2015; Di Yorio et al., 2016; Ogawa et al., 2016; Paullada-Salmerón et al., 2016a). GnIH neuronal fibers also innervate pituitary of teleost fishes (Ogawa and Parhar, 2014; Biswas et al., 2015; Ogawa et al., 2016; Paullada-Salmerón et al., 2016a). It was shown that GnIH fibers interact with GnRH-3 cell bodies in the preoptic area of zebrafish (Spicer et al., 2017).

Inhibitory effect of GnIH on the HPG axis was shown *in vivo* in European sea bass (Paullada-Salmerón et al., 2016b,c), goldfish (Zhang et al., 2010; Moussavi et al., 2012, 2013; Qi et al., 2013), cinnamon clownfish (Choi et al., 2016), orange-spotted grouper (Wang et al., 2015) and flatfish (Aliaga-Guerrero et al., 2017). However, stimulatory effect of GnIH on the HPG axis was also observed *in vivo* (Moussavi et al., 2012, 2013; Osugi et al., 2012; Wang et al., 2015; Paullada-Salmerón et al., 2016b). Both inhibitory and stimulatory effects of GnIH administration were observed on gonadotropin expression and release from cultured fish pituitaries (Amano et al., 2006; Shahjahan et al., 2011; Moussavi et al., 2012; Qi et al., 2013; Biran et al., 2014; Di Yorio et al., 2016; Spicer et al., 2017). On the other hand, forskolin-induced CRE-luciferase activity was suppressed by GnIH administration in COS-7 cells transfected with GnIH receptors in orange spotted grouper (Wang et al., 2015) and amphioxus (Osugi et al., 2014).

### Regulation of GH secretion

GnIH (RFRP-3) increases GH-releasing hormone mRNA expression in the hypothalamus and GH release in rats (Johnson et al., 2007; Johnson and Fraley, 2008). In bullfrog, LPXRFamide peptide (fGRP) also stimulates GH release (Koda et al., 2002). On the other hand, GnIH (LPXRFa peptide) has both stimulatory and inhibitory effects on GH release and/or expression in teleost fishes (Amano et al., 2006; Moussavi et al., 2014). Goldfish GnIH (gfLPXRFa) peptides stimulate GH release from cultured sockeye salmon pituitary cells (Amano et al., 2006). On the other hand, injection of gfLPXRFa to goldfish reduces basal serum GH levels but increases pituitary GH mRNA levels (Moussavi et al., 2014). Injection of gfLPXRFa reduces serum GH and pituitary GH mRNA levels stimulated by goldfish GnRH (sGnRH and cGnRH-II) (Moussavi et al., 2014). However, administration of gfLPXRFa to goldfish pituitary cells for 24-h generally increases basal GH release and attenuates sGnRH-induced changes in GH mRNA depending on the reproductive stage (Moussavi et al., 2014). These results indicate that the effect of GnIH (LPXRFa peptide) on GH release and/or expression depends on reproductive condition in teleost fishes.

### Regulation of cardiac contraction

Although expression levels of GnIH and GPR147 mRNAs are under detectable levels in the heart of rats, it was reported that human RFRP-1 and rat RFRP-1 rapidly and reversibly decrease the shortening and relaxation of cardiac myocytes in rats and rabbits (Nichols et al., 2010, 2012). Human RFRP-1 decreases the heart rate, stroke volume, ejection fraction, as well as cardiac output in mice (Nichols et al., 2010). It was suggested that human RFRP-1 impairs myocyte shortening by enhancing myofilament protein phosphorylation by protein kinase C (Nichols et al., 2012).

### Regulation of stress responses

Acute and chronic immobilization stress both up-regulates GnIH expression in the DMH of rats associated with inhibition of downstream HPG activity (Kirby et al., 2009). Endotoxin administration increases GnIH and GPR147 mRNA expression in rats (Iwasa et al., 2014). The effect of short-term fasting and high-fat diet on gonadotropin suppression was less effective in GPR147-deficient male mice, suggesting the involvement of GnIH-GPR147 pathway in the suppression of gonadotropin secretion by metabolic stress (León et al., 2014). Capture-handling stress and high ambient temperature increase GnIH expression in house sparrows (Calisi et al., 2008) and chicks (Chowdhury et al., 2012), respectively. Female presence also increases GnIH mRNA expression and GnIH release by rapid increase in norepinephrine in the PVN in male quail (Tobari et al., 2014). It was demonstrated that corticosterone increases GnIH mRNA expression *via* glucocorticoid receptor expressed in GnIH neurons in birds and mammals (Ahmed et al., 2014; Gojska and Belsham, 2014; Son et al., 2014). Neonatal dexamethasone exposure increases GnIH cell numbers in the DMH and GPR147 mRNA in the POA in female mice with delayed vaginal opening, irregular estrous cycles and lower GnRH expression in the POA (Soga et al., 2012). GnIH (RFRP) administration induces anxiety-related behavior in rats (Kaewwongse et al., 2011), suggesting that GnIH also mediates behavioral stress responses (Ubuka et al., 2018).

### Regulation of reproductive and aggressive behaviors

GnIH (RFRP-3) suppresses sexual behavior of male rats (Johnson et al., 2007) and proceptive sexual behavior and motivation of female hamsters (Piekarski et al., 2013). GnIH also inhibits copulation solicitation of female white-crowned sparrows exposed to male song (Bentley et al., 2006). RNA interference (RNAi) of the GnIH gene (GnIH RNAi) reduces resting time, spontaneous production of complex vocalizations, and enhances spontaneous brief agonistic vocalizations and song production of short duration in male birds when they were exposed to playbacks of novel male songs in white-crowned sparrows, suggesting that GnIH suppresses locomotor activity and aggressiveness (Ubuka et al., 2012b). GnIH directly activates P450arom and increases neuroestrogen synthesis in the brain and suppresses socio-sexual behavior of male birds (Ubuka et al., 2014).

### Regulation of feeding behavior

GnIH (RFRP-3) increases food intake in male rats (Johnson et al., 2007) and sheep (Clarke et al., 2012). GnIH mRNA levels are decreased in adult obese mice of both sexes (Poling et al., 2014). GnIH also stimulates food intake in chicks (Tachibana et al., 2005, 2008; McConn et al., 2014) and adult Pekin drakes (Fraley et al., 2013; see, Tsutsui and Ubuka, 2016) for a review). Studies in chicks suggested that the orexigenic effects of GnIH involves NPY, MCH, POMC neurons and opioid mu-receptor in the brain (Tachibana et al., 2008; McConn et al., 2014). Histological and physiological studies showed that NPY and POMC neurons are also regulated by GnIH in mammals (Qi et al., 2009; Ubuka et al., 2009b; Fu and van den Pol, 2010; Jacobi et al., 2013). The stimulatory effect of stress on GnIH neurons as well as the effect of GnIH on gonadotropin secretion, GH synthesis and release, and feeding activities suggest that GnIH neurones coordinate reproduction, growth and feeding activities in response to stress.

### Gonadal GnIH

GnIH and GPR147 exist in the testis of Syrian hamster (Zhao et al., 2010) and the ovary of mice (Singh et al., 2011) and humans (Oishi et al., 2012). GnIH (RFRP-3) suppresses spermatogenesis, follicular development and/or steroidogenesis in mice (Singh et al., 2011; Anjum et al., 2014), pigs (Zheng et al., 2015), and humans (Oishi et al., 2012).

GnIH and GPR147 also exist in the gonads and accessory reproductive organs in passeriform and galliform birds (Bentley et al., 2008; Maddineni S. R. et al., 2008; McGuire and Bentley, 2010; McGuire et al., 2011). GnIH suppresses testosterone secretion from the testis in house sparrow (McGuire and Bentley, 2010) and European starling (McGuire et al., 2011). Corticosterone up-regulates GnIH expression in the testis of European starlings, while metabolic stress up-regulates GnIH expression in the ovaries of European starlings (McGuire et al., 2013). Restrain stress also increases GnIH mRNA expression in the testes in zebra finches (Ernst et al., 2016).

### Evolutionary history of GnIH and its receptor

We previously searched for receptors that are structurally similar to GPR147 in the genome of mammals, birds, reptiles, amphibians, fishes, hemichordates, echinoderms, mollusks, insects, and cnidarians, to infer the evolutionary history of the GnIH system (Ubuka and Tsutsui, 2014). Neighbor joining (NJ) and maximum likelihood (ML) analyses of the amino acid sequences of the receptors grouped the receptors of vertebrates into GPR147 and GPR74. The receptors of insects were grouped into the receptor for SIFamide peptides that have a C-terminal YRKPPFNGSIFamide motif (Ubuka and Tsutsui, 2014). Human, quail, and zebrafish GPR147 was most structurally similar to SIFamide receptor within the C-terminal Famide peptide (SIFamide, FMRFamide, neuropeptide F, short neuropeptide F, drosulfakinin, myosuppressin) receptor families of fruit fly (Ubuka and Tsutsui, 2014). On the other hand, the amino acid sequences and the peptide coding regions of GnIH precursors were most similar to the FMRFamide precursor of fruit fly (Ubuka and Tsutsui, 2014). Chromosome synteny analysis of human, quail and zebrafish GnIH precursor genes and fruit fly Famide peptides precursor genes further identified a conserved synteny in vertebrate GnIH and fruit fly FMRFamide peptide precursor genes (Ubuka and Tsutsui, 2014). These results suggest that GnIH and its receptor pair have evolved from ancestral FMRFamide peptide and its receptor pair during the diversification and evolution of deuterostomian and protostomian species (Ubuka and Tsutsui, 2014).

Similarity-based clustering of various neuropeptide precursors in metazoan species forms one central cluster (Jékely, 2013). The core of the central cluster contains FMRFamide precursor of mollusk and nematode and GnIH precursor of vertebrates is evolutionarily directly related to FMRFamide precursor of mollusk and nematode (Jékely, 2013). FMRFamide-like peptides (FLPs) are the largest family of neuropeptides identified since the FMRFamide peptide was found in the venus clam *Macrocallista nimbosa* (Price and Greenberg, 1977; McVeigh et al., 2006). In order to further infer the evolutionary history of the GnIH-GnIH receptor system we investigate the structural similarities between GnIH and its receptor and well-studied nematode *Caenorhabditis elegans* (*C. elegans*) FLPs and their receptors and compare the functions of FLPs of nematode with GnIH of chordates.

## *C. elegans* FLP system

The sequencing of *C. elegans* genome (The *C. elegans* Sequencing Consortium, 1998) revealed 119 neuropeptide precursor genes which is subdivided into three major families, 31 *flp* gene family, 40 insulin-like peptide (*ins*) gene family, and 48 other neuropeptide-like protein (*nlp*) genes (Nelson et al., 1998; Li et al., 1999; Kim and Li, 2004; Frooninckx et al., 2012). Each FLP precursor gene of *C. elegans* encodes one to eight FLPs (Figure 1, Supplementary Figure 1, Table 2). *Flp*-29 and *flp-30* previously reported as parasite specific genes (McVeigh et al., 2006) are orthologous to *flp-28* and *flp-2*, respectively (McCoy et al., 2014). *Flp-31* is specific to plant parasitic nematodes (McCoy et al., 2014). Therefore, *C. elegans* do not have *flp-29, 30, 31* (Table 2). The mature sequences of 41 FLPs were biochemically identified within total 71 FLPs encoded in the 31 *flp* genes (McCoy et al., 2014; Peymen et al., 2014; Table 2). FMRFamide peptide is not encoded in *C. elegans flp* genes unlike that of mollusks. The relatedness of FLPs is still unclear because of the large diversity of the FLP sequences (Peymen et al., 2014). It was suggested that *flp-27* encodes C-terminal RXRFamide motif that is characteristic of NPF family of invertebrates (Clynen et al., 2009). FLPs are expressed in the majority of 302 neurons including sensory neurons, interneurons, and motor neurons in adult hermaphrodites and they are involved in neuroendocrine activity, locomotion, reproduction, and feeding (Peymen et al., 2014; Table 3). Predicted *C. elegans* neuropeptide receptors belong to rhodopsin and secretin GPCR families and 9 receptors that belong to rhodopsin family GPCR are activated by FLPs at EC_50_ of 1–100 nM (Frooninckx et al., 2012; Peymen et al., 2014; Table 3).

**Table 2:** *C. elegans fmrfamide-like peptide (flp)* genes and FMRFamide-like peptides (FLPs).

***flp* gene**	**FLPs**
*flp-1*	**SADPNFLRFa** (FLP-1-1), **SQPNFLRFa** (FLP-1-2), **ASGDPNFLRFa** (FLP-1-3), **SDPNFLRFa** (FLP-1-4), **AAADPNFLRFa** (FLP-1-5), KPNFLRFa (FLP-1-6), AGSDPNFLRFa (FLP-1-7), KPNFMRYa (FLP-1-8)
*flp-2*	**SPREPIRFa** (FLP-2-1), LRGEPIRFa (FLP-2-2)
*flp-3*	SPLGTMRFa (FLP-3-1), **TPLGTMRFa** (FLP-3-2), **EAEEPLGTMRFa** (FLP-3-3), NPLGTMRFa (FLP-3-4), **ASEDALFGTMRFa** (FLP-3-5), **SAEPFGTMRFa** (FLP-3-7), **SADDSAPFGTMRFa** (FLP-3-8), **NPENDTPFGTMRFa** (FLP-3-9)
*flp-4*	PTFIRFa (FLP-4-1), ASPSFIRFa (FLP-4-2)
*flp-5*	**GAKFIRFa** (FLP-5-1), AGAKFIRFa (FLP-5-2), APKPKFIRFa (FLP-5-3)
*flp-6*	**KSAYMRFa** (FLP-6) × 6
*flp-7*	**SPMQRSSMVRFa** (FLP-7-1) × 3, **TPMQRSSMVRFa** (FLP-7-2) × 2, SPMERSAMVRFa (FLP-7-3), SPMDRSKMVRFa (FLP-7-4)
*flp-8*	**KNEFIRFa** (FLP-8) × 3
*flp-9*	**KPSFVRFa** (FLP-9) × 2
*flp-10*	QPKARSGYIRFa (FLP-10)
*flp-11*	**AMRNALVRFa** (FLP-11-1), **ASGGMRNALVRFa** (FLP-11-2), **NGAPQPFVRFa** (FLP-11-3)
*flp-12*	RNKFEFIRFa (FLP-12)
*flp-13*	**AMDSPLIRFa** (FLP-13-1), **AADGAPLIRFa** (FLP-13-2), **APEASPFIRFa** (FLP-13-3) × 2, **ASPSAPLIRFa** (FLP-13-4), **SPSAVPLIRFa** (FLP-13-5), ASSAPLIRFa (FLP-13-6), **SAAAPLIRFa** (FLP-13-7)
*flp-14*	**KHEYLRFa** (FLP-14) × 4
*flp-15*	**GGPQGPLRFa** (FLP-15-1), **RGPSGPLRFa** (FLP-15-2)
*flp-16*	**AQTFVRFa** (FLP-16-1) × 2, **GQTFVRFa** (FLP-16-2)
*flp-17*	KSAFVRFa (FLP-17-1) × 3, KSQYIRFa (FLP-17-2)
*flp-18*	DFDGAMPGVLRFa (FLP-18-1), EMPGVLRFa (FLP-18-2), SVPGVLRFa (FLP-18-3) × 3, EIPGVLRFa (FLP-18-4), SEVPGVLRFa (FLP-18-5), DVPGVLRFa (FLP-18-6)
*flp-19*	**WANQVRFa** (FLP-19-1), **ASWASSVRFa** (FLP-19-2)
*flp-20*	AMMRFa (FLP-20) × 2
*flp-21*	GLGPRPLRFa (FLP-21)
*flp-22*	**SPSAKWMRFa** (FLP-22) × 3
*flp-23*	VVGQQDFLRFa (FLP-23)
*flp-24*	**VPSAGDMMVRFa** (FLP-24)
*flp-25*	DYDFVRFa (FLP-25-1), **ASYDYIRFa** (FLP-25-2)
*flp-26*	**EFNADDLTLRFa** (FLP-26-1), **GGAGEPLAFSPDMLSLRFa** (FLP-26-2)
*flp-27*	GLGGRMRFa (FLP-27)
*flp-28*	**APNRVLMRFa** (FLP-28)
*flp-32*	AMRNSLVRFa (FLP-32)
*flp-33*	**APLEGFEDMSGFLRTIDGIQKPRFa** (FLP-33)
*flp-34*	ALNRDSLVASLNNAERLRFa (FLP-34)

*Biochemically identified mature peptides are shown in bold (Peymen et al., 2014). Peptide names are from Krajniak (2013). Numbers of peptides are shown if the same peptide is encoded in the precursor*.

**Table 3:** Deorphanized *C. elegans* FLP receptors and their putative roles.

**Receptors**	**FLPs**	**Putative roles**	**References**
NPR-1	21	Feeding behavior, Thermal avoidance, Ethanol tolerance, Innate immunity, Regulation of aggregation, Aerotaxis	de Bono and Bargmann, 1998; Kubiak et al., 2003; Rogers et al., 2003, 2006; Davies et al., 2004; Gray et al., 2004; Cheung et al., 2005; Gloria-Soria and Azevedo, 2008; Styer et al., 2008; Macosko et al., 2009; Glauser et al., 2011; Milward et al., 2011; Jang et al., 2012
NPR-3	15-1, 15-2	Locomotion	Keating et al., 2003
NPR-4	1-6, 4-2, 18-2, 18-5	Fat storage, Olfaction, Foraging, Reproduction	Keating et al., 2003; Cohen et al., 2009
NPR-5a, NPR-5b	18-1, 18-2, 18-3, 18-4, 18-5, 18-6	Fat storage, Dauer formation	Kubiak et al., 2008; Cohen et al., 2009
NPR-10a, NPR-10b	3-1, 3-3, 3-5, 3-7, 3-8		
NPR-11	21	Local search behavior, Olfactory adaptation, Reproduction	Keating et al., 2003; Chalasani et al., 2010
FRPR-18a, FRPR-18b	2-1		
NPR-22a, NPR-22b	7-3		
EGL-6a, EGL-6b	10, 17-1, 17-2	Inhibition of egg laying	Ringstad and Horvitz, 2008

*Only FLPs that bind receptors at nanomolar range are shown Frooninckx et al., 2012; Peymen et al., 2014*.

## Evolutionary history of chordate GNIH, NPFF and *C. elegans* FLP precursors and peptides

A multiple sequence alignment of the protein sequences of human, Japanese quail, fire belly newt, coelacanth, zebrafish, spotted gar, lamprey, amphioxus GnIH (RFRP, LPXRFa) peptide, and *C. elegans* FLP precursors was performed by EMBL-EBI Clustal Omega Multiple Sequence Alignment software (Figure 1, Supplementary Figure 1). GnIH (RFRP, LPXRFa) peptides of human, Japanese quail, fire belly newt, coelacanth, zebrafish, spotted gar aligned into five groups named from the N-terminal of their precursors, which are RFRP-1/GnIH-RP-1/LPXRFa-1, RFRP-2/GnIH/LPXRFa-2, RFRP-3/LPXRFa-3, GnIH-RP-2/LPXRFa-4, and LPXRFa-5 peptide groups (Figure 1, Supplementary Figure 1). The RFRP-1/GnIH-RP-1/LPXRFa-1 group consists of human RFRP-1, quail GnIH-RP-1, newt nLPXRFa-2, coelacanth LPXRFa-1, zebrafish LPXRFa-1, and gar LPXRFa-1, which all peptides have LPLRFamide sequence at their C-termini (Figure 1, Supplementary Figure 1, Table 1). The RFRP-2/GnIH/LPXRFa-2 group consists of human RFRP-2, quail GnIH, coelacanth LPXRFa-2, and gar LPXRFa-2, which only quail GnIH and gar LPXRFa-2 have LPLRFamide sequence at their C-termini (Figure 1, Supplementary Figure 1, Table 1). Human RFRP-2 and coelacanth LPXRFa-2 have C-terminal LPLRSamide and LPLRLamide sequences, respectively (Figure 1, Supplementary Figure 1, Table 1). The RFRP-3/LPXRFa-3 group consists of human RFRP-3, newt LPXRFa-1, coelacanth LPXRFa-3, zebrafish LPXRFa-2, and gar LPXRFa-3, which all peptides have LPQRFamide sequence at their C-termini (Figure 1, Supplementary Figure 1, Table 1). A peptide that has a sequence of APNLSNRSamide can be predicted to be produced from quail GnIH precursor, which aligns to this peptide group (Figure 1, Supplementary Figure 1, Table 1). The GnIH-RP-2/LPXRFa-4 group consists of quail GnIH-RP-2, newt LPXRFa-3, coelacanth LPXRFa-4, zebrafish LPXRFa-3, which all peptides also have LPQRFamide sequence at their C-termini (Figure 1, Supplementary Figure 1, Table 1). The LPXRFa-5 group only consists of newt LPXRFa-4 and coelacanth LPXRFa-5, which both peptides have LPQRFamide sequence at their C-termini (Figure 1, Supplementary Figure 1, Table 1). Lamprey LPXRFa-1a and amphioxus PQRFa-2 align to the RFRP-1/GnIH-RP-1/LPXRFa-1 group peptides, but these peptides have C-terminal PQRFamide sequence instead of LPLRFamide sequence (Figure 1, Supplementary Figure 1, Table 1). Lamprey lLPXRFa-2 and amphioxus PQRFa-3 align to the GnIH-RP-2/LPXRFa-4 group peptides which have C-terminal LPQRFamide sequence (Figure 1, Supplementary Figure 1, Table 1).

Twenty one, fourteen, thirteen, sixteen, and five *C. elegans* FLPs align to RFRP-1/GnIH-RP-1/LPXRFa-1, RFRP-2/GnIH/LPXRFa-2, RFRP-3/LPXRFa-3, GnIH-RP-2/LPXRFa-4, and LPXRFa-5 peptide groups, respectively (Figure 1, Supplementary Figure 1, Table 2). However, the C-terminal sequences of the aligned FLPs were not identical besides the RFamide sequence. There were twenty-four FLPs that did not align to either groups (Figure 1, Supplementary Figure 1, Table 2).

**Figure 3:**
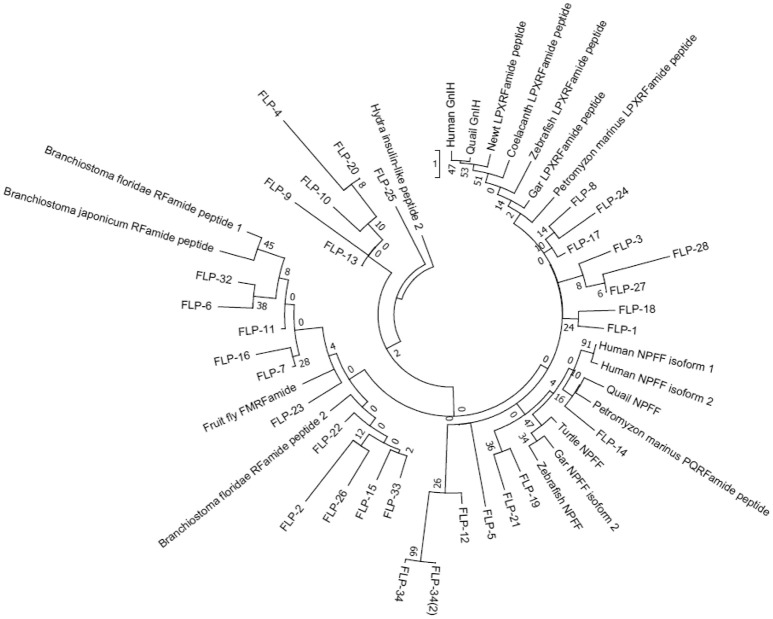
A phylogenetic analysis of human, quail, newt, coelacanth, zebrafish, gar, lamprey, amphioxus GnIH, human, quail, turtle, zebrafish, gar, lamprey, amphioxus NPFF, fruit fly FMRFamide and *C. elegans* FMRFamide-like peptide (FLP) precursors. Human, quail, newt, coelacanth, zebrafish, gar, lamprey, amphioxus GnIH, human, quail, turtle, zebrafish, gar, lamprey, amphioxus NPFF, fruit fly FMRFamide, and *C. elegans* FLP precursor polypeptides were aligned by CLUSTALW Multiple Sequence Alignment. Multiple alignment parameters were as follows: Gap open penalty 10, Gap extension penalty 0.2, Protein weight matrix GONNET with residue-specific and hydrophilic penalties. Molecular phylogenetic analysis was performed by Maximum Likelihood method using MEGA7 (Kumar et al., 2016). The Maximum Likelihood method was based on the JTT matrix-based model (Jones et al., 1992). The tree with the highest log likelihood is shown. Initial tree for the heuristic search were obtained automatically by applying Neighbor-Join and BioNJ algorithms to a matrix of pairwise distances estimated by using a JTT model, and then selecting the topology with superior log likelihood value. The tree is drawn with branch lengths measured in the number of substitutions per site. The analysis involved 51 amino acid sequences. All positions containing gaps and missing data were eliminated. There were a total of 32 positions in the final dataset. The phylogeny was tested by 50 Bootstrap replications. Accession numbers are human (*Homo sapiens*) GnIH precursor (Human GnIH; NP_071433.3), Japanese quail (*Coturnix japonica*) GnIH precursor (Quail GnIH; XP_015709159.1), Japanese fire belly newt (*Cynops pyrrhogaster*) GnIH precursor (Newt LPXRFamide peptide; BAJ78290.1), West Indian Ocean coelacanth (*Latimeria chalumnae*) GnIH precursor (Coelacanth LPXRFamide peptide; XP_005993154.1), zebrafish (*Danio rerio*) GnIH precursor (Zebrafish LPXRFamide peptide, NP_001076418.1), spotted gar (*Lepisosteus oculatus*) GnIH precursor (Gar LPXRFamide peptide; XP_015213317.1), sea lamprey (*Petromyzon marinus*) GnIH precursor (Petromyzon marinus LPXRFamide peptide; BAL52329.1), Japanese amphioxus (*Branchiostoma japonicum*) GnIH precursor (Branchiostoma japonicum RFamide peptide; BAO77760.1), human NPFF precursor isoform 1 (Human NPFF isoform 1; NP_003708.1), human NPFF precursor isoform 2 (Human NPFF isoform 2; NP_001307225.1), Japanese quail NPFF precursor (Quail NPFF; XP_015705838.1), Western painted turtle (*Chrysemys picta bellii*) NPFF precursor (Turtle NPFF; XP_005307776.1), zebrafish NPFF precursor (Zebrafish NPFF; BAF34891.1), spotted gar NPFF precursor isoform X2 (Gar NPFF isoform 2; XP_015199730.1), sea lamprey NPFF precursor (Petromyzon marinus PQRFamide peptide; BAE79779.1), Florida lancelet (*Branchiostoma floridae*) RFamide precursor 1 (Branchiostoma floridae RFamide peptide 1; XP_002599251.1), Florida lancelet RFamide precursor 2 (Branchiostoma floridae RFamide peptide 2; XP_002609543.1), Fruit fly (*Drosophila melanogaster*) FMRFamide precursor (Fruit fly FMRFamide; NP_523669.2), *C. elegans* (*Caenorhabditis elegans*) FLP-1 precursor (AAC46464.1), FLP-2 precursor (NP_001024945.1), FLP-3 precursor (AAC08940.1), FLP-4 precursor (AAC08941.1), FLP-5 precursor (AAC08942.1), FLP-6 precursor (AAC08943.1), FLP-7 precursor (AAC08944.1), FLP-8 precursor (AAC08945.1), FLP-9 precursor (AAC08946.1), FLP-10 precursor (AAC08947.1), FLP-11 precursor (NP_001024752.1), FLP-12 precursor (AAC08950.1), FLP-13 precursor (AAC08951.1), FLP-14 precursor (NP_499682.2), FLP-15 precursor (NP_499820.1), FLP-16 precursor (NP_001022091.1), FLP-17 precursor (NP_503051.1), FLP-18 precursor (NP_508514.2), FLP-19 precursor (NP_509776.1), FLP-20 precursor (NP_509574.2), FLP-21 precursor (NP_505011.2), FLP-22 precursor (NP_492344.2), FLP-23 precursor (AAY18633.1), FLP-24 precursor (AAW78866.1), FLP-25 precursor (NP_001022665.1), FLP-26 precursor (NP_741827.1), FLP-27 precursor (NP_495111.1), FLP-28 precursor (NP_001024947.1), FLP-32 precursor (NP_510551.2), FLP-33 precursor (NP_871818.1), FLP-34 precursor isoform 1 (FLP-34; NP_001300170.1), FLP-34 precursor isoform 2 (FLP-34'; NP_503365.1). Hydra (*Hydra vulgaris*) insulin-like peptide 2 precursor sequence (Hydra insulin-like peptide 2; ADA67986.1) served as outgroup (root) of the tree.

We next performed a phylogenetic analysis of human, quail, newt, coelacanth, zebrafish, gar, lamprey, amphioxus GnIH, human, quail, turtle, zebrafish, gar, lamprey, amphioxus NPFF, fruit fly FMRFamide, and *C. elegans* FLP precursors (Figure 3, Supplementary Figure 2). Molecular phylogenetic analysis was performed by ML method using MEGA7 (Kumar et al., 2016). The subclades of human, quail, newt, coelacanth, zebrafish, gar, lamprey GnIH (Osugi et al., 2012, LPXRFamide), and human, quail, turtle, zebrafish, gar, lamprey NPFF (Osugi et al., 2006, PQRFamide) precursors formed a larger clade with FLP-1, 3, 5, 8, 12, 14, 17, 18, 19, 21, 24, 27, 28, 34 (Figure 3). Amphioxus RFamide peptide precursors and fruit fly FMRFamide precursors formed a different clade with FLP-2, 6, 7, 11, 15, 16, 22, 23, 26, 32, 33 (Figure 3). These results suggest that GnIH (LPXRFamide peptide) and NPFF (PQRFamide peptide) precursor genes of vertebrates are diverged from their common ancestral gene by gene duplication (Osugi et al., 2006, 2012, 2014).

**Figure 4:**
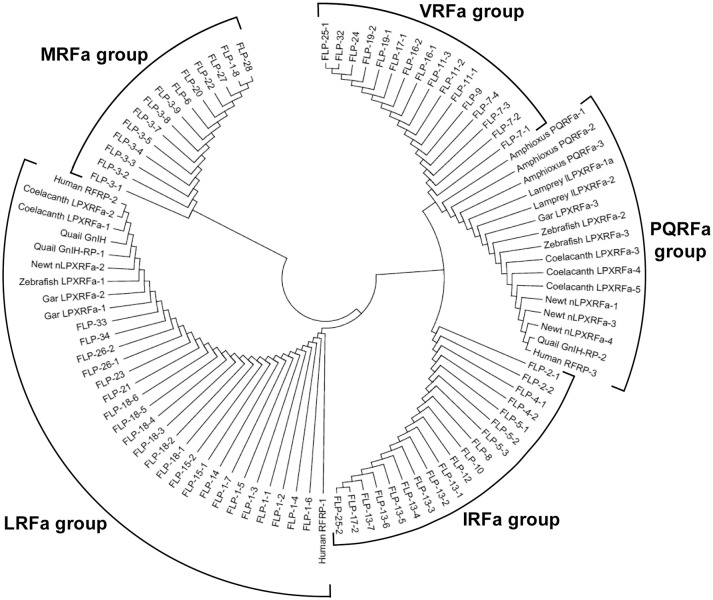
A phylogenetic analysis of human, quail, newt, coelacanth, zebrafish, gar, lamprey, and amphioxus GnIH (LPXRFa) peptides and *C. elegans* FMRFamide-like peptides (FLPs). Predicted or biochemically identified endogenous peptides shown in Table 3 were analyzed. The accession numbers of their precursor proteins are shown in the legend of Figure 1. The phylogenetic tree was constructed by Neighbor Joining method (Saitou and Nei, 1987) with the proportion of different sites statistical substitution model using MEGA 7 (Kumar et al., 2016).

We then investigated the structural similarity of the predicted or biochemically identified human, Japanese quail, fire belly newt, coelacanth, zebrafish, spotted gar, lamprey, and amphioxus GnIH (RFRP, LPXRFamide) peptides and *C. elegans* FLPs by using NJ method (Saitou and Nei, 1987). This evolutionary analyses were also conducted using an online software MEGA7 (Kumar et al., 2016). Interestingly, 97 peptides analyzed were grouped into five groups that have characteristic C-terminals, MRFa, LRFa, VRFa, PQRFa, and IRFa (Figure 4, Table 4). Gar LPXRFa-1 and 2, zebrafish LPXRFa-1, coelacanth LPXRFa-1 and 2, newt nLPXRFa-2, quail GnIH and GnIH-RP-1, human RFRP-1 and RFRP-2 were grouped in the LRFa group with FLP-1-1, 2, 3, 4, 5, 6, 7, FLP-14, FLP-15-1, 2, FLP-18-1, 2, 3, 4, 5, 6, FLP-21, FLP-23, FLP-26-1, 2, FLP-33, and FLP-34 (Figure 4, Table 4). Amphioxus PQRFa-1, 2, 3, lamprey lLPXRFa-1a, 2, gar LPXRFa-3, zebrafish LPXRFa-2, 3, coelacanth LPXRFa-3, 4, 5, newt nLPXRFa-1, 3, 4, quail GnIH-RP-2 and human RFRP-3 formed a single group evolutionarily related to FLPs in the VRFa and IRFa groups (Figure 4, Table 4). FLPs produced from the same precursor were generally grouped in the same peptide groups because of the same or similar C-terminal amino acid sequences (Table 4). The exceptions are FLP-1-8 (MRFa group) and other FLP-1 peptides (LRFa group) encoded in *flp-1*, FLP-17-1 (VRFa group) and FLP-17-2 (IRFa group) encoded in *flp-17*, and FLP-25-1 (VRFa group) and FLP-25-2 (IRFa group) encoded in *flp-25* (Tables 2, 4).

**Table 4:** Grouping of GnIH (LPXRFa) peptides of chordates and FLPs of *C. elegans*.

**MRFa GROUP**	
FLP-1-8	KPNF**MRYa**
FLP-3-1	SPLGT**MRFa**
**FLP-3-2**	TPLGT**MRFa**
**FLP-3-3**	EAEEPLGT**MRFa**
FLP-3-4	NPLGT**MRFa**
**FLP-3-5**	ASEDALFGT**MRFa**
**FLP-3-7**	SAEPFGT**MRFa**
**FLP-3-8**	SADDSAPFGT**MRFa**
**FLP-3-9**	NPENDTPFGT**MRFa**
**FLP-6**	KSAY**MRFa**
FLP-20	AM**MRFa**
**FLP-22**	SPSAKW**MRFa**
FLP-27	GLGGR**MRFa**
**FLP-28**	APNRVL**MRFa**
**LRFa GROUP**	
**FLP-1-1**	SADPNF**LRFa**
**FLP-1-2**	SQPNF**LRFa**
**FLP-1-3**	ASGDPNF**LRFa**
**FLP-1-4**	SDPNF**LRFa**
**FLP-1-5**	AAADPNF**LRFa**
FLP-1-6	KPNF**LRFa**
FLP-1-7	AGSDPNF**LRFa**
**FLP-14**	KHEY**LRFa**
**FLP-15-1**	GGPQGP**LRFa**
**FLP-15-2**	RGPSGP**LRFa**
FLP-18-1	DFDGAMPGV**LRFa**
FLP-18-2	EMPGV**LRFa**
FLP-18-3	SVPGV**LRFa**
FLP-18-4	EIPGV**LRFa**
FLP-18-5	SEVPGV**LRFa**
FLP-18-6	DVPGV**LRFa**
FLP-21	GLGPRP**LRFa**
FLP-23	VVGQQDF**LRFa**
**FLP-26-1**	EFNADDLT**LRFa**
**FLP-26-2**	GGAGEPLAFSPDMLS**LRFa**
**FLP-33**	APLEGFEDMSGFLRTIDGIQKP**RFa**
FLP-34	ALNRDSLVASLNNAER**LRFa**
Gar LPXRFa-1	LYHSVTNLP**LRFa**
Gar LPXRFa-2	ASQPVANLP**LRFa**
Zebrafish LPXRFa-1	PAHLHANLP**LRFa**
Coelacanth LPXRFa-1	FSNSVINLP**LRFa**
Coelacanth LPXRFa-2	LSQSLANLP**LRLa**
**Newt nLPXRFa-2**	MPHASANLP**LRFa**
**Quail GnIH**	SIKPSAYLP**LRFa**
Quail GnIH-RP-1	VPNSVANLP**LRFa**
**Human RFRP-1**	MPHSFANLP**LRFa**
Human RFRP-2	SAGATANLP**LRSa**
**FLP-7-1**	SPMQRSSM**VRFa**
**FLP-7-2**	TPMQRSSM**VRFa**
FLP-7-3	SPMERSAM**VRFa**
FLP-7-4	SPMDRSKM**VRFa**
**FLP-9**	KPSF**VRFa**
**FLP-11-1**	AMRNAL**VRFa**
**FLP-11-2**	ASGGMRNAL**VRFa**
**FLP-11-3**	NGAPQPF**VRFa**
**FLP-16-1**	AQTF**VRFa**
**FLP-16-2**	GQTF**VRFa**
FLP-17-1	KSAF**VRFa**
**FLP-19-1**	WANQ**VRFa**
**FLP-19-2**	ASWASS**VRFa**
**FLP-24**	VPSAGDMM**VRFa**
FLP-25-1	DYDF**VRFa**
FLP-32	AMRNSL**VRFa**
**PQRFa GROUP**	
**Amphioxus PQRFa-1**	WDEAWR**PQRFa**
**Amphioxus PQRFa-2**	GDHTKDGWR**PQRFa**
**Amphioxus PQRFa-3**	GRDQGWR**PQRFa**
**Lamprey lLPXRFa-1a**	SGVGQGRSSKTLFQ**PQRFa**
**Lamprey lLPXRFa-2**	SEPFWHRTR**PQRFa**
Gar LPXRFa-3	AALNL**PQRFa**
Zebrafish LPXRFa-2	STINL**PQRFa**
Zebrafish LPXRFa-3	SGTGPSATL**PQRFa**
Coelacanth LPXRFa-3	IPMAIPNL**PQRFa**
Coelacanth LPXRFa-4	SFMQPLANL**PQRFa**
Coelacanth LPXRFa-5	FIQSVANL**PQRFa**
**Newt nLPXRFa-1**	SVPNL**PQRFa**
**Newt nLPXRFa-3**	SIQPLANL**PQRFa**
**Newt nLPXRFa-4**	APSAGQFIQTLANL**PQRFa**
**Quail GnIH-RP-2**	SSIQSLLNL**PQRFa**
**Human RFRP-3**	VPNL**PQRFa**
**IRFa GROUP**	
**FLP-2-1**	SPREP**IRFa**
FLP-2-2	LRGEP**IRFa**
FLP-4-1	PTF**IRFa**
FLP-4-2	ASPSF**IRFa**
**FLP-5-1**	GAKF**IRFa**
FLP-5-2	AGAKF**IRFa**
FLP-5-3	APKPKF**IRFa**
**FLP-8**	KNEF**IRFa**
FLP-10	QPKARSGY**IRFa**
FLP-12	RNKFEF**IRFa**
**FLP-13-1**	AMDSPL**IRFa**
**FLP-13-2**	AADGAPL**IRFa**
**FLP-13-3**	APEASPF**IRFa**
**FLP-13-4**	ASPSAPL**IRFa**
**FLP-13-5**	SPSAVPL**IRFa**
FLP-13-6	ASSAPL**IRFa**
**FLP-13-7**	SAAAPL**IRFa**
FLP-17-2	KSQY**IRFa**
**FLP-25-2**	ASYDY**IRFa**

*Biochemically identified mature peptide names are shown in bold (Peymen et al., 2014; Ubuka et al., 2016). Characteristic C-terminal sequences that are identical within each group are also shown in bold*.

## Evolutionary history of human GNIH and NPFF receptors and *C. elegans* FLP receptors

**Figure 5:**
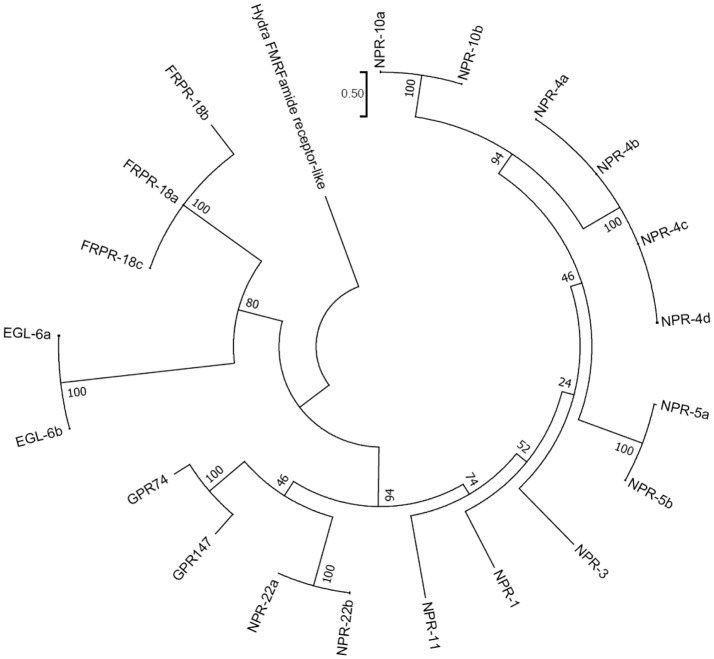
A phylogenetic analysis of human GnIH receptors (GPR147, GPR74) and *C. elegans* FMRFamide-like peptide (FLP) receptors. Human GnIH receptors (GPR147, GPR74) and *C. elegans* FMRFamide-like peptide (FLP) receptor amino acid sequences were aligned by CLUSTALW Multiple Sequence Alignment. Multiple alignment parameters were Gap open penalty 10, Gap extension penalty 0.2, Protein weight matrix GONNET with residue-specific and hydrophilic penalties. Molecular phylogenetic analysis was performed by Maximum Likelihood method using MEGA7 (Kumar et al., 2016). The Maximum Likelihood method was based on the JTT matrix-based model (Jones et al., 1992). The tree with the highest log likelihood is shown. Initial tree for the heuristic search were obtained automatically by applying Neighbor-Join and BioNJ algorithms to a matrix of pairwise distances estimated by using a JTT model, and then selecting the topology with superior log likelihood value. The tree is drawn with branch lengths measured in the number of substitutions per site. The analysis involved 21 amino acid sequences. All positions containing gaps and missing data were eliminated. There were a total of 282 positions in the final dataset. The phylogeny was tested by 50 Bootstrap replications. The accession numbers of human GnIH receptors are GPR147 (NPFF1; NP_071429.1), GPR74 (NPFF2; AAG41398.1). WormBase IDs of *C. elegans* FLP receptors are NPR-1 (WP:CE06941), NPR-3 (WP:CE08056), NPR-4 isoform a (WP:CE37317), NPR-4 isoform b (WP:CE50076), NPR-4 isoform c (WP:CE50063), NPR-4 isoform d (WP:CE50035), NPR-5a (WP:CE33345), NPR-5b (WP:CE36962), NPR-10a (WP:CE19767), NPR-10b (WP:CE36989), NPR-11 (WP:CE47199), FRPR-18 isoform a (WP:CE28679), FRPR-18 isoform b (WP:CE29349), FRPR-18 isoform c (WP:CE52203), NPR-22 isoform a (WP:CE31260), NPR-22 isoform b (WP:CE38456), EGL-6a (WP:CE04219), EGL-6b (WP:CE43400). Predicted hydra (*Hydra vulgaris*) FMRFamide receptor-like protein sequence (Hydra FMRFamide receptor-like; XP_012564736.1) served as outgroup (root) of the tree.

A recent phylogenomic study of GnRH receptors identified that GnIH/NPFF receptors have representation in both bilaterian and non-bilaterian lineages (Plachetzki et al., 2016) suggesting that GnIH/NPFF receptors evolved before the divergence of bilaterian and non-bilaterian species. In this study, we investigated the evolutionary history of human GnIH receptors (GPR147, GPR74) and *C. elegans* FLP receptors by ML method using MEGA7 (Kumar et al., 2016; Figure 5, Supplementary Figure 3). GPR147 and GPR74 were suggested to have strong evolutionary relationships with neuropeptide receptor (NPR)-22a and 22b, followed by NPR-11, NPR-1, NPR-3, NPR-5a and 5b, NPR-4a, 4b, 4c, 4d, NPR-10a and 10b (Figure 5). EGL-6a and 6b, FRPR-18a, 18b, 18c formed a different clade (Figure 5).

## Roles of *C. elegans* FLPS and their receptors

NPR-1 is the first GPCR that was deorphanized in *C. elegans* (Kubiak et al., 2003; Rogers et al., 2003). It was shown that NPR-1 is involved in various functions, such as feeding behavior, thermal avoidance, ethanol tolerance, and innate immunity (de Bono and Bargmann, 1998; Davies et al., 2004; Gray et al., 2004; Cheung et al., 2005; Rogers et al., 2006; Gloria-Soria and Azevedo, 2008; Styer et al., 2008; Glauser et al., 2011; Milward et al., 2011; Jang et al., 2012; Table 3). NPR-1 signaling occurs through G_α*i*_ (Kubiak et al., 2003; Rogers et al., 2003). NPR-1 was suggested to suppress aggregation by inhibiting RMG neuron that is a hub of gap junction network that connects sensory neurons (Macosko et al., 2009). The RMG neuron connects five sensory neurons that are known to trigger aggregation. NPR-1 inhibits this gap junction driven activation of RMG neuron. Deletion of NPR-1 increases the threshold for heat avoidance involving RMG neuron (Glauser et al., 2011; Jang et al., 2012). NPR-1 also suppresses aerotaxis behavior in the presence of food by inhibiting ERX, AQR, PQR, and SDQ neurons (Cheung et al., 2005; Chang et al., 2006). NPR-1 binds FLP-21 with high affinity (Table 3).

It was shown that FLPs encoded by *flp*-18 are potent ligands of NPR-5a and NPR-5b, which are the splice variants of *npr-5* (Kubiak et al., 2008; Cohen et al., 2009; Table 3). NPR-4 is also activated by FLP-18 peptides (Cohen et al., 2009; Table 3). *Flp-18* loss of function or *npr-4* and *npr-5* deletion mutants display dauer formation, foraging defects, accumulation of excess intestinal fats and reduce aerobic metabolism (Cohen et al., 2009). It is hypothesized that detection of nutrition by sensory neurons (AWC, AFD, ASE) induces FLP-18 peptides release from AIY interneurons. FLP-18 peptides induces fat accumulation by acting on NPR-4 in intestine and NPR-5 in ciliated sensory neurons. NPR-4 in RIV and AVA neurons modulates responses to odor and foraging behavior. FLP-18 peptides also regulate dauer formation by acting on NPR-5 in ASJ neurons (Cohen et al., 2009).

Two GPCR isoforms EGL-6a and EGL-6b inhibit egg-laying (Ringstad and Horvitz, 2008). FLP-10, FLP-17-1, and FLP-17-2 activate EGL-6 and inhibit egg-laying via G_α*i*_ (Ringstad and Horvitz, 2008). Unfavorable conditions stimulate the release of FLP-17-1 and FLP-17-2 from BAG neurons, which inhibit HSN motor neurons *via* EGL-6. HSN motor neurons stimulate vulval muscles that are involved in egg-laying (Trent et al., 1983; White et al., 1986). FLP-10 release from vulva and spermatheca also inhibits egg-laying *via* EGL-6 (Kim and Li, 2004; Ringstad and Horvitz, 2008).

NPR-3 RNAi significantly impairs locomotion and some animals will be paralyzed. Green fluorescent protein reporter construct for NPR-3 indicated that NPR-3 is expressed in all excitatory and inhibitory motor neurons that have their cell bodies in the ventral nerve cord (Keating et al., 2003). NPR-3 binds FLP-15-1, 2 with high affinities (Table 3). RNAi for NPR-4 and NPR-11 significantly decreases the number of egg laid (Keating et al., 2003). NPR-4 binds FLP-1-6, FLP-4-2, FLP-18-2, 5 with high affinities (Table 3). On the other hand, NPR-11 binds FLP-21 with a high affinity (Table 3).

## Conserved function of GNIH, FLPS, and their receptors

As summarized earlier GnIH regulates reproductive activity in chordates by acting on GnRH neurons in the brain or gonadotropes in the pituitary or within the gonads (Hinuma et al., 2000; Tsutsui et al., 2000; Fukusumi et al., 2001; Ukena et al., 2002, 2003b; Bentley et al., 2003, 2008; Ubuka et al., 2003, 2005, 2006, 2008, 2009b,c, 2012a, 2013; Yano et al., 2003, 2004; Yoshida et al., 2003; Ciccone et al., 2004; Osugi et al., 2004, 2012; Amano et al., 2006; Kriegsfeld et al., 2006; Shimizu and Bédécarrats, 2006, 2010; Johnson et al., 2007; Clarke et al., 2008, 2012; Dardente et al., 2008; Gibson et al., 2008; Johnson and Fraley, 2008; Maddineni S. et al., 2008; Maddineni S. R. et al., 2008; Murakami et al., 2008; Revel et al., 2008; Small et al., 2008; Smith et al., 2008, 2010, 2012; Anderson et al., 2009; Ducret et al., 2009; Gingerich et al., 2009; Joseph et al., 2009; Kadokawa et al., 2009; Legagneux et al., 2009; Qi et al., 2009, 2013; Rizwan et al., 2009, 2012; Sari et al., 2009; Wu et al., 2009; Chowdhury et al., 2010; Mason et al., 2010; McGuire and Bentley, 2010; Pineda et al., 2010a,b; Quennell et al., 2010; Sethi et al., 2010; Tobari et al., 2010; Zhang et al., 2010; Zhao et al., 2010, 2014; McGuire et al., 2011; Molnár et al., 2011; Shahjahan et al., 2011; Singh et al., 2011; Iwasa et al., 2012; Li et al., 2012; Losa-Ward et al., 2012; Moussavi et al., 2012, 2013, 2014; Oishi et al., 2012; Poling et al., 2012, 2013; Son et al., 2012, 2016; Fraley et al., 2013; Harbid et al., 2013; Henson et al., 2013; Janati et al., 2013; Klosen et al., 2013; Salehi et al., 2013; Anjum et al., 2014; Biran et al., 2014; Glanowska et al., 2014; Gojska et al., 2014; Ikeno et al., 2014; Jafarzadeh Shirazi et al., 2014; Jørgensen et al., 2014; León et al., 2014; Ogawa and Parhar, 2014; Piekarski et al., 2014; Sáenz de Miera et al., 2014; Soga et al., 2014; Biswas et al., 2015; Russo et al., 2015; Semaan and Kauffman, 2015; Surbhi et al., 2015; Wang et al., 2015; Xiang et al., 2015; Zheng et al., 2015; Choi et al., 2016; Di Yorio et al., 2016; Ogawa et al., 2016; Paullada-Salmerón et al., 2016a,b,c; Aliaga-Guerrero et al., 2017; Muñoz-Cueto et al., 2017; Spicer et al., 2017). It is interesting that many of *C. elegans* FLP receptors such as NPR-4, NPR-11, EGL-6 and their ligands are involved in the regulation of reproductive activities (Table 3). It is especially interesting that EGL-6a and EGL-6b activated by FLP-10, FLP-17-1, and FLP-17-2 inhibit egg-laying *via* G_α*i*_ (Ringstad and Horvitz, 2008), which is analogous to inhibition of reproductive activities by GnIH *via* GPR147 in vertebrates. Expression of GnIH or GnIH neuronal activity is stimulated by stress and hence it is thought that GnIH mediates the inhibitory effects of stress on reproduction (Calisi et al., 2008; Kirby et al., 2009; Chowdhury et al., 2012; Soga et al., 2012; McGuire et al., 2013; Ahmed et al., 2014; Gojska and Belsham, 2014; Iwasa et al., 2014; Son et al., 2014; Tobari et al., 2014; Ernst et al., 2016). It was also shown in *C. elegans* that unfavorable conditions stimulate the release of FLP-17-1 and FLP-17-2, which inhibit egg-laying *via* EGL-6 (Trent et al., 1983; White et al., 1986; Kim and Li, 2004; Ringstad and Horvitz, 2008). Thus, mediation of the inhibitory effect of stress on reproductive activities may be a conserved property of GnIH and FLP systems.

GnIH is further hypothesized to be a general mediator of behavioral stress responses as GnIH suppresses locomotor activity, aggression, and reproductive behavior *via* GPR147 (Liu et al., 2001; Bentley et al., 2006; Kaewwongse et al., 2011; Ubuka et al., 2012b, 2014, 2018; Piekarski et al., 2013). NPR-1 activated by FLP-21 is involved in thermal avoidance behavior, ethanol tolerance and suppresses aggregation through G_α*i*_ (de Bono and Bargmann, 1998; Kubiak et al., 2003, 2008; Rogers et al., 2003, 2006; Davies et al., 2004; Gray et al., 2004; Cheung et al., 2005; Chang et al., 2006; Gloria-Soria and Azevedo, 2008; Styer et al., 2008; Macosko et al., 2009; Glauser et al., 2011; Milward et al., 2011; Jang et al., 2012; Table 3), which is analogous to the action of GnIH mediating the effect of stress on behavior (Ubuka et al., 2018). NPR-3 activated by FLP-15-1, FLP-15-2 also regulates locomotion (Keating et al., 2003; Table 3).

GnIH stimulates feeding behavior in rats (Johnson et al., 2007), sheep (Clarke et al., 2012), chicks (Tachibana et al., 2005, 2008; McConn et al., 2014), and Pekin drakes (Fraley et al., 2013) and GnIH mRNA expression is reduced in adult obese mice (Poling et al., 2014; see Tsutsui and Ubuka, 2016 for a review). Loss of function of FLP-18 or its receptors NPR-4, NPR-5 induces foraging defects, accumulation of excess intestinal fats and reduction in aerobic metabolism (Kubiak et al., 2008; Cohen et al., 2009; Table 3). Regulation of feeding behavior and metabolism may also be a conserved property of GnIH and FLPs, although the precise mechanism is not understood.

## Conclusion

**Figure 6:**
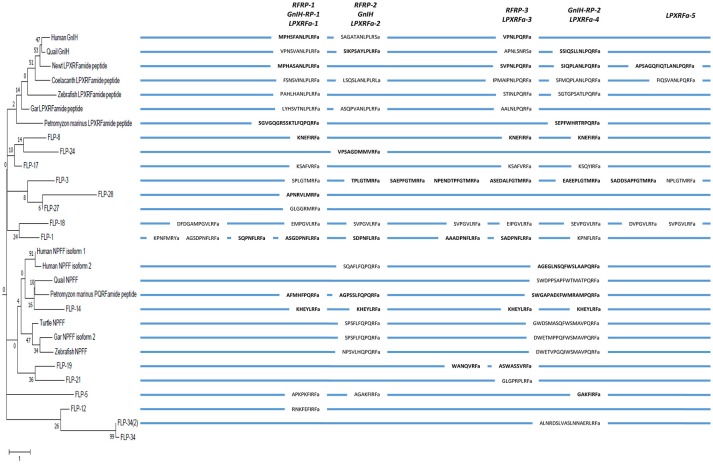
A schematic representation of the multiple sequence alignment of human, quail, newt, coelacanth, zebrafish, gar, lamprey GnIH, human, quail, turtle, zebrafish, gar, lamprey NPFF, and *C. elegans* FMRFamide-like peptide (FLP) precursors highlighting the sequences of identified and predicted biologically active peptides. Each precursor is shown next to the branch of the partial phylogenetic tree of human, quail, newt, coelacanth, zebrafish, gar, lamprey, amphioxus GnIH, human, quail, turtle, zebrafish, gar, lamprey, amphioxus NPFF, fruit fly FMRFamide and *C. elegans* FLP precursors (Figure 3). Note that the alignment of the peptides is based on multiple sequence alignment of all precursors (Supplementary Figure 2). Therefore, human NPSF (NPFF) is aligned to human RFRP-2 instead of human RFRP-1 (Figure 2).

In order to infer the evolutionary history of the GnIH-GnIH receptor system, we compared the structures and functions of GnIH and its receptor of chordates with *C. elegans* FLPs and their receptors. One or two C-terminal LPLRFamide peptides and one to three C-terminal LPQRFamide peptides were encoded in the LPXRFamide (X = L or Q) precursor polypeptide genes of jawed vertebrates (human, quail, newt, coelacanth, zebrafish, gar). Orthologous LPXRFamide precursor polypeptide genes of lamprey and amphioxus encoded only two or three C-terminal PQRFamide peptides. Each FLP precursor gene encodes one to eight FLPs that have generally the same C-terminal sequences especially the last three amino acids. A multiple sequence alignment and phylogenetic analyses of GnIH, NPFF and FLP precursors (Figures 3, 6, Supplementary Figure 2) have shown that GnIH and NPFF precursors belong to different clades and there are FLPs that have structural similarities to either precursor. FLP-1, 3, 8, 17, 18, 24, 27, and 28 precursors form a clade with GnIH precursors, while FLP-14, 19, and 21 precursors form a different clade with NPFF precursors (Figures 3, 6). Although the peptide coding regions of FLP precursors in the same clade align well with those of GnIH or NPFF precursors, the sequence similarities of the peptides within the aligned precursors were weak (Figure 6). On the other hand, alignment of GnIH (LPXRFa) peptides of chordates and FLPs of *C. elegans* grouped the peptides into five groups according to the last C-terminal amino acid sequences, which were MRFa, LRFa, VRFa, IRFa, and PQRFa. C-terminal LPLRFamide peptides of jawed vertebrates were all in the LRFa group with other FLPs. On the other hand, C-terminal LPQRFamide peptides of jawed vertebrates and C-terminal PQRFamide peptides of lamprey and amphioxus were grouped in the PQRFa group excluding FLPs. C-terminal LPLRFamide peptides may be the original form of LPXRFamide (X = L or Q) peptides as many FLPs have the C-terminal LRFa sequence.

Phylogenetic analysis of GnIH receptors and FLP receptors suggested that GPR147 and GPR74 have a strong evolutionary relationship with NPR-22, followed by NPR-11, NPR-1, NPR-5, NPR-4, and NPR-10. It is interesting that these receptors regulate reproduction, locomotion and feeding as GnIH and GPR147. It is also important that NPR-11 and NPR-3 bind FLP-21 and FLP-15-1,-2, respectively, peptides which all have a C-terminal PLRFamide sequence. GnIH and some FLPs mediate the effect of stress on reproduction and behavior, which may also be a conserved property of these peptide systems. Future studies are needed to investigate how neuropeptide precursor genes are mutated to evolve new neuropeptides and their inheritance.

## Author contributions

All authors listed have made a substantial, direct and intellectual contribution to the work, and approved it for publication.

### Conflict of interest statement

The authors declare that the research was conducted in the absence of any commercial or financial relationships that could be construed as a potential conflict of interest.
