# Supplementary material for: Comparative and Evolutionary Aspects of Gonadotropin-Inhibitory Hormone and FMRFamide-Like Peptide Systems
Source: Front Neurosci. 2018 Oct 18;12:747. doi: 10.3389/fnins.2018.00747 (PMC6200920; doi:10.3389/fnins.2018.00747)
Supplement: Supplementary file 1 [file Table_1.DOCX]

**SUPPLEMENTARY FIGURE 1 | A multiple sequence alignment of human, quail, newt, coelacanth, zebrafish, gar, lamprey, amphioxus GnIH and *C. elegans* FMRFamide-like peptide (FLP) precursors.** Human, quail, newt, coelacanth, zebrafish, gar, lamprey, amphioxus GnIH and *C. elegans* FLP precursor polypeptides were aligned by EMBL-EBI Clustal Omega Multiple Sequence Alignment software. Identified or predicted endogenous peptide sequences are underlined. Biochemically identified endogenous peptide sequences are shown in bold. Glycine (G) is an amidation signal. Lysine (K) and/or arginine (R) as endoproteolytic basic amino acids are Italicized. Accession numbers are human (*Homo sapiens*) GnIH precursor (NP_071433.3), Japanese quail (*Coturnix japonica*) GnIH precursor (XP_015709159.1), Japanese fire belly newt (*Cynops pyrrhogaster*) GnIH precursor (BAJ78290.1), West Indian Ocean coelacanth (*Latimeria chalumnae*) GnIH precursor (XP_005993154.1), zebrafish (*Danio rerio*) GnIH precursor (NP_001076418.1), spotted gar (*Lepisosteus oculatus*) GnIH precursor (XP_015213317.1), sea lamprey (*Petromyzon marinus*) GnIH precursor (BAL52329.1), Japanese amphioxus (*Branchiostoma japonicum*) GnIH precursor (BAO77760.1), *C. elegans* (*Caenorhabditis elegans*) FLP-1 precursor (AAC46464.1), FLP-2 precursor (NP_001024945.1), FLP-3 precursor (AAC08940.1), FLP-4 precursor (AAC08941.1), FLP-5 precursor (AAC08942.1), FLP-6 precursor (AAC08943.1), FLP-7 precursor (AAC08944.1), FLP-8 precursor (AAC08945.1), FLP-9 precursor (AAC08946.1), FLP-10 precursor (AAC08947.1), FLP-11 precursor (NP_001024752.1), FLP-12 precursor (AAC08950.1), FLP-13 precursor (AAC08951.1), FLP-14 precursor (NP_499682.2), FLP-15 precursor (NP_499820.1), FLP-16 precursor (NP_001022091.1), FLP-17 precursor (NP_503051.1), FLP-18 precursor (NP_508514.2), FLP-19 precursor (NP_509776.1), FLP-20 precursor (NP_509574.2), FLP-21 precursor (NP_505011.2), FLP-22 precursor (NP_492344.2), FLP-23 precursor (AAY18633.1), FLP-24 precursor (AAW78866.1), FLP-25 precursor (NP_001022665.1), FLP-26 precursor (NP_741827.1), FLP-27 precursor (NP_495111.1), FLP-28 precursor (NP_001024947.1), FLP-32 precursor (NP_510551.2), FLP-33 precursor (NP_871818.1), FLP-34 precursor isoform 1 (FLP-34; NP_001300170.1), FLP-34 precursor isoform 2 (FLP-34’; NP_503365.1).

**Supplementary Figure 1-1**

Human -------------------------------------------------------MEIIS

Japanese quail -------------------------------------------------------MEIIS

Fire belly newt -------------------------------------------------------MEILL

Coelacanth ------------------------------------------------------------

Zebrafish ------------------------------------------------------------

Spotted gar -------------------------------------------------------MTPNS

Lamprey -----------------------------------------------------------M

Amphioxus -------------------------------------------------------MRTLV

FLP-1 --MTLLYQVGLLLLVA---AT-------------------YKVSA---ECCTPGATSDF-

FLP-2 ----------------------------------------------------------MQ

FLP-3 -------------------------------------------------------MISPN

FLP-4 -------------------------------------------------------MNAF-

FLP-5 ------------MRSV---PA-------------------FQLPRQHPPFTKQSFLATM-

FLP-6 -----------------------------------------------------------M

FLP-7 -----------------------------------------------------MLGSRF-

FLP-8 ------MLSGVLFSIF-VLAI-SANAS---C-----------DVSALTTENEKE------

FLP-9 MCVYVCAQTPP----IRVLSILSQDSAPIKAHFFFWSRFQRKTQQHRLKKGETFFVSKKK

FLP-10 ----------------------------------------------------------MQ

FLP-11 ----------------------------------------------------------MT

FLP-12 ------------------------------------------------------------

FLP-13 -------------------------------------------------------MMT--

FLP-14 --MMICLPTALLLSAF-VVAA-SGQEAPAGAGAS-----GAAQAPHNPKDCQAILANNGD

FLP-15 --------------------------------------------------------MQF-

FLP-16 ------------------------------------------------------MNFSGF

FLP-17 ------MLSKLVLTTCLLLTI-SGSSQAASM-----------EEIQSEKFCEKF------

FLP-18 -------------------------------------------------------MQRW-

FLP-19 -------------------------------------------------------MSFQ-

FLP-20 -------------------------------------------------------MLGYT

FLP-21 ------------------------------------------------------------

FLP-22 -------------------------------------------------------MNR--

FLP-23 -----------------------------------------------------MLLPKIS

FLP-24 -----------------------------------------------------MLSSRT-

FLP-25 -------------------------------------------------------MSHNS

FLP-26 ------------------------------------------------------------

FLP-27 -------------------------------------------------------MFSLT

FLP-28 -------------------------------------------------------MFSVR

FLP-32 -------------------------------------------------------MLSFV

FLP-33 ------------------------------------------------------------

FLP-34 ------------------------------------------------------------

FLP-34’ ------------------------------------------------------------

Human -SKL---FILLTLATSSLLTSNIFCADELVMS----------------------NLHSKE

Japanese quail -TQK---FILLTLATVAFLTPHGACLDELMKS----------------------SLESRE

Fire belly newt -MSR---LLLLTLATLGLA-SQILGLEDPGRS----------------------HLYGEE

Coelacanth -----------------------MPISEPPTI---------------------------E

Zebrafish -MSY---FALLSLALGILS-SFMLSEVTALRL----------------------PLSGER

Spotted gar --CW---PVLLLLGCSVLQTPAAQSADERPLS----------------------A-----

Lamprey -LAGFL--LLHCLHFAFVA---PY-------PESAHGSPLENQALQYLTEDDALLI---R

Amphioxus -VLTWISTLFPFLLAA------T-----------ATGS-------------DPRT-----

FLP-1 -CTVF--SMLSTMEQNE-----------VMNFIGENCD--GDAEVALQKME*KR*--KPNFM

FLP-2 -VSGILSALFLVLLAVIVS---PF-------QFVQPK-----RILPIPTSRDQLLR---G

FLP-3 HLILL-----FCVNCAFLVASDAT-------P*KR*--------------------------

FLP-4 -SSSLKTFIFSLLFATLL---ALTAAHP--PSSGEEIAEQEEKNIAS-------------

FLP-5 -SSRS-TTIAFLFIATLL---VFQCVSA--QSSAEDADYLE-------------------

FLP-6 NSRGL-----ILTLGVVIAVAFAQ-------QDSEVER-------EMM*KR***KSAYMRFG***R*S

FLP-7 -L--L--LALGLLVLVLA---EE-SAEQQVQ---------EPTEL-----EKSGEQLSEE

FLP-8 --------LGLRICHLEAEMQVVQ---RAL---QE--------VMQ----QTDVTL----

FLP-9 KMNQFY-ALFLVACIAAMANAYEE---PDLDALAEFCGKESNRKYC----DQIAQL----

FLP-10 -LSIVFVFFVLCLAAVF----------------------------AVPISDASRAR---R

FLP-11 QFSAL-----ALLLIVFVAASFAQ-------SYDDVSA-------E*KR***AMRNALVRFG***R*-

FLP-12 -MNVQV-IIALLFCLIATCATQKVKGSPEVLPAAMYDGELSHESVNKISAQLLNAL----

FLP-13 -------SLLT----ISMFVVAIQAFDSSEI--------------RMLDEQYDTKNPFFQ

FLP-14 QQE-------ALLCQLSES-SMLL---AQL---GA--------LVS----EGVERL----

FLP-15 -STLI--RVAVFAVLAIA---TLADYDD----------------------NSVGTIPVAV

FLP-16 EFSSIV-AFFLLILQLSTA-AVLP---ADY---AY--------GVA----DEMSAL----

FLP-17 --------PTLHMCRLKEELTGSL---VEL---QY--------LLQ----DGINNQ----

FLP-18 -SGVL--LI-SLCCLLRG---AL-AYTEPIYEIVEEDIPAEDIEVTRTNEKQDGRVFS*KR*

FLP-19 -LTLF--SMLFLLIAVV-----------VGQPI---QS--QNGDLKMQAVQDNSPLNMEA

FLP-20 QSRVV---ITLLLFSVFLAVCMAT-------PSGYPGQ-------ELQNVSDDYPIYEEE

FLP-21 -MRLF--ILLSCLLAWV-----------LAAPY---ID--QEDALRVLN------AYLEQ

FLP-22 -------SMIALCVVLMVSLVSAQVFDLDGQ--------------QLAG-----------

FLP-23 -ILL---YILVV-----L------------------------------------------

FLP-24 -SSII--LILAILVAIMA---VA-QCRN----------------I-----QY--------

FLP-25 MIYLLV-AFLVLLCAT-----TEAKKECSI------------------DCQEDGSA----

FLP-26 -MKVM--FMLALLFSSLV---ATSAFRLPFQFFGAN----EDFNSGLTKRNYYESKPY*KR*

FLP-27 QILTF-----LLVAITLMTFSSAQ-------PIDEERP-------IFMERREA-SAFGDI

FLP-28 SIFAI-----FCVLILALSTINA-------------------------------------

FLP-32 QTLIL-----ALLCSIVFVE--AM-------PSM--RP-------A*KK*------------

FLP-33 --MR---FLILIVAIVLLSAVHGFSVEPRLAA------------------------FADG

FLP-34 -MHSLI-TELLIFFTVLLSVSVLS---LPL------------------------------

FLP-34’ -MQFQF-LMALIFVALVLTDSVLS---LPL------------------------------

**Supplementary Figure 1-2**

Human NYD-KYSEPR--G-YPKGERSLNFEEL-------------KDWGPKNVIKMSTPAVN*K***MP**

Japanese quail DDDDKYYEIKDSI-LEEKQRSLNFEEM-------------KDWGSKNFMKVNTPTVN*K*VP

Fire belly newt GDEEDFSESNEDI-FEETQRSANSGEE-------------KEVGVRNMVKMSAPLVH*R***MP**

Coelacanth VVTKVFQEASQES-LEERQRSLSNEQL-------------KEWEPKATIKMKTPIIS*K*FS

Zebrafish DLNGFTWGQFSEN-AQEIPRSLEIQDFT--L----NVAPTSGGASSPTILRLHPIIP*K*PA

Spotted gar DQDRLDPDATHEN-MQEELRSIELERI-------------QDILPSVTRKIDVPTIQ*K*LY

Lamprey ------KLQSRMNTALEGAMVEEVEDCG--VAATDEVQGSRENRAAL----*R***SGVGQGRS**

Amphioxus -------------------------TYK--VS*R*---------**WDEAWRPQRFG***R*SG*R***GDH**

FLP-1 RYG*R*SAAVKSLG*KK*AGSDPNFLRFG*R*----------------------**SQPNFLRFG***K*--

FLP-2 ------QLAYLKGTTVAQPAV------------NDNTLGIFEAS-------------AMA

FLP-3 ------------------------------------------------------------

FLP-4 ----------------PDELIPEIVEQQ------------NFWPPVH-------LRGLRS

FLP-5 ------------------------------------------------------KYQRIA

FLP-6 ------D----------------GGN------------------------------PMEM

FLP-7 DLI---D--EQ*KR***TPMQRSSMVRFG***R*---------------------------------**S**

FLP-8 ----------------Y-----------------------D-------------QEVPVM

FLP-9 ----------------ATQHAIG----I------------N-------------QEQVRM

FLP-10 ------QVASE----------------------------------------------*KR*-

FLP-11 ----------------------------------------------------------**AS**

FLP-12 ----------------SELEALQ-EGNQ------------Q-------------LKMAE*K*

FLP-13 FLENSKR--SDRPT*R***AMDSPLIRFG***KR*-------------------**AADGAPLIRFG***R*--

FLP-14 ----------------VQTHGLALEEET------------N-------------EGDNDM

FLP-15 DLD---Y-------------F-------------------------S-------NYV*K*--

FLP-16 ----------------PD------------------------------------SGS---

FLP-17 ----------------Q-----------------------Q-------------AGAQEV

FLP-18 ------------DFDGAMPGVLRFG*KR*G------------GVWEKRE-------SSVQ--

FLP-19 -FNDDSAL--YDYLEQSDPSLKSME*KR*---------------------------------

FLP-20 GLQLSAE-GTDEPH-EEKRAVFRMG*K*------------------------------*R*AMM

FLP-21 -FGPGSDR--VYYVAEDDH--GSM*K*-----------------------------------

FLP-22 -LEQN------------DARLMEQQV*KR*-------------------**SPSAKWMRFG***K*--

FLP-23 ---------------------------------------------------------QET

FLP-24 ------D--VEEMTPEAAFRYAQWGEIP--------------------------------

FLP-25 ------------------------------------------------------------

FLP-26 -------------**EFNADDLTLRFG***KR*---------------------------------

FLP-27 -------------------------I------------------------------GELK

FLP-28 ------------------------------------------------------------

FLP-32 ------------------------------------------------------------

FLP-33 GAAELAQEARQ-----A--RNAELEFIKRFLPAKE*RR*---------**APLEGFEDMSGFLR**

FLP-34 ------------------------E*KK*-------------------------ADISTFAS

FLP-34’ ------------------------E*KK*-------------------------ADISTFAS

***RFRP-1/GnIH-RP-1/LPXRFa-1 RFRP-2/GnIH/LPXRFa-2***

Human **HSFANLPLRFG***R*NVQEE*R*---------------------------SAGATANLPLR-SG*R*

Japanese quail NSVANLPLRFG*R*SNPEE*R*---------------------------**SIKPSAYLPLR**-**FG***R*

Fire belly newt **HASANLPLRFG***R*AFLEEAKSSP---------------------------AFYSPLR-YES

Coelacanth NSVINLPLRFG*R*AFPDG*R*---------------------------LSQSLANLPLR-LG*R*

Zebrafish HLHANLPLRFG*R*DAQPGTG-----------------------------------------

Spotted gar HSVTNLPLRFG*R*---------------------------------ASQPVANLPLR-FG*R*

Lamprey **SKTLFQPQRFG***R*GVPPPAADCPESA----AASWAGLQDGNADRAS*R*--------------

Amphioxus **TKDGWRPQRFG***R*------------------------------------------------

FLP-1 **ASGDPNFLRFG***R*-------------------------------------**SDPNFLR**-**FG***K*

FLP-2 ------------------------------------------------------------

FLP-3 --SPLGTMRFG*KR*AIADEMTFEEDGYYPSNVMWKRSTVDSSEPVIRDQ*R***TPLGTMR**-**FG***K*

FLP-4 SNG*K*PTFIRFG*KR*------------------------------------ASPSFIR-FG*K*

FLP-5 *R*APKPKFIRFG*R*-------------------------------------AGAKFIR-FG*R*

FLP-6 E*KR***KSAYMRFG***KR*SSGGDEQE-----LV----------GGDDID--ME*KR***KSAYMR**-**FG***K*

FLP-7 **PMQRSSMVRFG***KR*---------------------------------**SPMQRSSMVR**-**FG***K*

FLP-8 N*KR***KNEFIRFG***KR*SDG-----------------------------------------M--

FLP-9 E*KR***KPSFVRFG***KR*SGYPLVIDD------------------------------EEMR-M--

FLP-10 ------------------------------------------------------------

FLP-11 **GGMRNALVRFG***KR*SPLDEEDF----------------APESPLQG*KR***NGAPQPFVR**-**FG***R*

FLP-12 *R*RNKFEFIRFG-------------------------------------------------

FLP-13 **APEASPFIRFG***KR*----------------------------------AADGAPLIR-FG*R*

FLP-14 E*KR***KHEYLRFG***KR*-------------------------------------**KHEYLR**-**F**--

FLP-15 *K***GGPQGPLRFG***KR*-----------------------------------**RGPSGPLR**-**FG***K*

FLP-16 ---LFAEQRPS*KR*-------------------------------------**AQTFVR**-**F**--

FLP-17 Q*KR*KSAFVRFG*KR*SAP----EE------------------------------EAME-M--

FLP-18 *KK*EMPGVLRFG*KR*AYFDE*KK*-----------------------------SVPGVLR-FG*K*

FLP-19 ------------------------------------------------------------

FLP-20 RFG*KR*AMMRFG*KR*SVFRLG-----------------------------------------

FLP-21 ------------------------------------------------------------

FLP-22 *R***SPSAKWMRFG***K*------------------------------------------------

FLP-23 AAVRGALFRSGRAVPFE*R*-----------------------------VVGQQDFLR-FG*R*

FLP-24 -H*K*---------*R*---------------------------------**VPSAGDMMVR**-**FG***K*

FLP-25 ------AVDLGLVLPPELYEST------------------------------RLSNLLAR

FLP-26 ---------------------------------------**GGAGEPLAFSPDMLSLR**-**FG***K*

FLP-27 G*K*GLGGRMRFG*KR*SSSPDISLAEMRAIY----------GGDQSN---------IFN-FK-

FLP-28 -**APNRVLMRFG***KR*GGNSEGHLGY-RFVPAGAPAIAEYIDVDDVIG-------GDDR-F--

FLP-32 -AMRNSLVRFG*KR*ADPVGTDD-----VF----------LGE-----SYGSADPYEY-VPE

FLP-33 **TIDGIQKPRFG**-------------------------------------------------

FLP-34 AINNAGRLRYG*KR*SDPAMWEEN------------------------------NVII-PSS

FLP-34’ AINNAGRLRYG*KR*SDPAMWEEN------------------------------NVII-PSS

**Supplementary Figure 1-3**

***RFRP-3/LPXRFa-3***

Human NMEVSLV------------------------------*RR***VPNLPQRFG***R*TTT--AKSVC-

Japanese quail AFGESLS------------------------------*RR*APNLSNRSG*R*SPL—A*R*-----

Fire belly newt AFDERI*R*------------------------------*K***SVPNLPQRFG***R*YLA—S*KR*----

Coelacanth ALEN*R*------------------------------IPMAIPNLPQRFG*R*SPL—-V*K----*

Zebrafish ---DRAP------------------------------*K*STINLPQRFG*R*SCTMCA*R*----

Spotted gar GLTEGSA---------------------------RKA*K*AALNLPQRFG*R*APA--R---L-

Lamprey ------------------------------------------------------------

Amphioxus ------------------------------------------------------------

FLP-1 -------------------**AAADPNFLRFG***KR*------**SADPNFLRFG***R*SF---DNFDR-

FLP-2 ------------------------------------------------------------

FLP-3 *R***SAEPFGTMRFG***KR*--**NPENDTPFGTMRFG***KR*---**ASEDALFGTMRFG***KR*EDGNAPFGTM

FLP-4 ------------------------------------------------------------

FLP-5 S-------------RN-----------------TWEDGYASPSVN---------ELY---

FLP-6 *R*SGPQEDDM------PME*K*--*R***KSAYMRFG***KR*SSDMEV---IGNEGVDG--DAHDLF---

FLP-7 *R*-----------------**SPMQRSSMVRFG***KR*----SPMERSAMVRFG*R*-----------

FLP-8 -----------------------------E*KR*--------**KNEFIRFG***K*-----------

FLP-9 -----------------------------D*KR*--------**KPSFVRFG***RK*----------

FLP-10 ------------------------------------------------------------

FLP-11 SGQ--------------------------------------------LD--HMHDLL---

FLP-12 ------------------------------------------------------------

FLP-13 -------------------**APEASPFIRFG***KR*-----**ASPSAPLIRFG***R*-----------

FLP-14 -----------------------------**G***KR*--------**KHEYLRFG***K*-----------

FLP-15 *R*-------------SSFHVAPAAEDVASWYQ-----------------------------

FLP-16 -----------------------------**G***KR*--------**AQTFVRFG***K*-----------

FLP-17 -----------------------------E*KR*--------KSAFVRFG*R*SFGM-EPQIT-

FLP-18 *R*-------------SYFDE*KK*SVPGVLRFG*KR*DVPMD*KR*EIPGVLRFG*KR*DYMADSFD*K*-

FLP-19 ----------------------**WANQVRFG***KR*------**ASWASSVRFG**------------

FLP-20 ------------------------------------------------------------

FLP-21 -------------------------------*R*------GLGPRPLRFG------------

FLP-22 *R*-------------------**SPSAKWMRFG***KR*SGAEA-----VSE---------------

FLP-23 AGMASGV----------------------GGGSEGGPDDVKNSYIRVNGEPEIV------

FLP-24 *R*-------------SI--------------------------------------------

FLP-25 P-------------SSQ----------FKM*KR*--------DYDFVRFG*R*AAPI-------

FLP-26 ------------------------------------------------------------

FLP-27 ------------------------------------------------------------

FLP-28 ------------------------------------------------------------

FLP-32 RMSNR----------------GPSSVLLY-------------------------------

FLP-33 ------------------------------------------------------------

FLP-34 E-------------DQY----------LYSEGR--------YPYALI*KR*--------ALN

FLP-34’ E-------------DQY----------LYSEGR--------YPYALI*KR*--------ALN

***GnIH-RP-2/LPXRFa-4***

Human ------RMLSDLCQGSMHSPCANDLFYSMTC-----------------------------

Japanese quail ---**SSIQSLLNLPQRFG***K*S-----VPISLSQ-----------------------------

Fire belly newt ----**SIQPLANLPQRFG***R*--------------------------------**APSAGQFIQT**

Coelacanth ---SFMQPLANLPQRFG*R*SPFYD*K*--------------------------------FIQS

Zebrafish ---SGTGPSATLPQRFG*RR*NIFALDPLRALA-----------------------------

Spotted gar ------PPLPAVPQRAVYAPVEEDEKSSQEL-----------------------------

Lamprey ---**SEPFWHRTRPQRFG***KR*GGDPAS-------PM--------------------------

Amphioxus -----**GRDQGWRPQRFG***R*TEAGLREVLGGEAFPLLQMTRTDLHDDLPAMAVRYTPPAARL

FLP-1 ------ES*R*KPNFLRFG*K*------------------------------------------

FLP-2 ------*KR*LRGEPIRFG*KR***SPREPIRFG***KR*FNPLPD---YDFQ-----------------

FLP-3 KFG*KR***EAEEPLGTMRFG***KR***SADDSAPFGTMRFG**----*KR*---------------------

FLP-4 ------------------------------------------------------------

FLP-5 ------V*KR***GAKFIRFG**-------------------------------------------

FLP-6 -------*KR***KSAYMRFG***KR*SMGEEEDHDMM*K*--------*R*--------------------

FLP-7 -----SPMDRSKMVRFG*R*SSIDR---ASMVRLG----*KR*------------------**TPM**

FLP-8 --------*R***KNEFIRFG***R*SDKGL---G----LD----D-NDVSSEF--------------

FLP-9 ------------------------------------------------------------

FLP-10 -----QPKARSGYIRFG*KR*RVD----------PNAE---LLYLDQLLI------------

FLP-11 -------STLQ-KLKFANNK----------------------------------------

FLP-12 -----------------*RK*-----------------------------------------

FLP-13 ------**SPSAVPLIRFG***R*-----**SAAAPLIRFG**----*R*---------------------A

FLP-14 --------*R***KHEYLRFG***RK*-----------------------------------------

FLP-15 ------------------------------------------------------------

FLP-16 --------*R***GQTFVRFG***R*SAPFE---Q---------------------------------

FLP-17 ------E*KR*KSQYIRFG*K*------------------------------------------

FLP-18 ------*R*SEVPGVLRFG*KR*-----DVPGVLRFG----*KR*SDLEEHYAGVLL*KK--*-----

FLP-19 ------------------------------------------------------------

FLP-20 ------------------------------------------------------------

FLP-21 ------------------------------------------------------------

FLP-22 -------------QDY--------------------------------------------

FLP-23 ------------------------------------------------------------

FLP-24 ------------------------------------------------------------

FLP-25 ------*KK***ASYDYIRFG***RK*-----------------------------------------

FLP-26 ------------------------------------------------------------

FLP-27 ------------------------------------------------------------

FLP-28 ------------------------------------------------------------

FLP-32 ------------------------------------------------------------

FLP-33 ------------------------------------------------------------

FLP-34 RDSLVASLNNAERLRFG*RK*-----------------------------------------

FLP-34’ RDSLVASLNNAERLRFG*RK*-----------------------------------------

**Supplementary Figure 1-4**

***LPXRFa-5***

Human ---QHQEIQNP-DQK---------------------QSR------RLLFKKIDDAELKQE

Japanese quail ---GVQE-SEP-GM----------------------------------------------

Fire belly newt **LANLPQRFG***R*SIDLHKLCNF--------ANTYAKGGQES------GYGDKRMLDADNGPE

Coelacanth VANLPQRFG*R*SPSVSNYPHS--------TVAFPVQ--FE------RYQQTN---------

Zebrafish ---LYTRTPESPSFPK-----------------ERTQVH------DYMFETVEDSEETVK

Spotted gar ------------------------------------------------------------

Lamprey ------------------------------------------------------------

Amphioxus RALPLLRLYDRGALSQLINGPPKQPATNREVYPPSLRMIRAAAEGLRGFAHQQDKDTG--

FLP-1 ------------------------------------------------------------

FLP-2 ------------------------------------------------------------

FLP-3 NPLGTMRFG*K*--------------------------------------------------

FLP-4 ------------------------------------------------------------

FLP-5 ------------------------------------------------------------

FLP-6 -**KSAYMRFG***R*--------------------------------------------------

FLP-7 **QRSSMVRFG***KR*SMEF-----------------EM-----------QSNEKNIEDSE----

FLP-8 -------FGYTSDVF-----------------YL--------------------------

FLP-9 ------------------------------------------------------------

FLP-10 ------------------------------------------------------------

FLP-11 ------------------------------------------------------------

FLP-12 ------------------------------------------------------------

FLP-13 SSAPLIRFG*RK*-------------------------------------------------

FLP-14 ------------------------------------------------------------

FLP-15 ------------------------------------------------------------

FLP-16 ------------------------------------------------------------

FLP-17 ------------------------------------------------------------

FLP-18 SVPGVLRFG*RK*-------------------------------------------------

FLP-19 ------------------------------------------------------------

FLP-20 ------------------------------------------------------------

FLP-21 ------------------------------------------------------------

FLP-22 ------------------------------------------------------------

FLP-23 ----YQ------------------------------------------------------

FLP-24 ------------------------------------------------------------

FLP-25 ------------------------------------------------------------

FLP-26 ------------------------------------------------------------

FLP-27 ------------------------------------------------------------

FLP-28 ------------------------------------------------------------

FLP-32 ------------------------------------------------------------

FLP-33 ------------------------------------------------------------

FLP-34 ------------------------------------------------------------

FLP-34’ ------------------------------------------------------------

Human K------------------------------ 196

Japanese quail ------------------------------- 173

Fire belly newt EEQKEEGATQNNWNQNHNQMVM--------- 233

Coelacanth ------------------------------- 172

Zebrafish NT---------DYTALD-------------- 198

Spotted gar ------------------------------- 157

Lamprey ------------------------------- 159

Amphioxus -ESFAPPRSNDDWLAEIQRLGLRGRKRRDVS 208

FLP-1 ------------------------------- 175

FLP-2 ------------------------------- 106

FLP-3 ------------------------------- 184

FLP-4 ------------------------------- 93

FLP-5 ------------------------------- 116

FLP-6 ------------------------------- 170

FLP-7 ------------------------------- 177

FLP-8 ------------------------------- 135

FLP-9 ------------------------------- 159

FLP-10 ------------------------------- 67

FLP-11 ------------------------------- 110

FLP-12 ------------------------------- 85

FLP-13 ------------------------------- 160

FLP-14 ------------------------------- 143

FLP-15 ------------------------------- 87

FLP-16 ------------------------------- 92

FLP-17 ------------------------------- 130

FLP-18 ------------------------------- 208

FLP-19 ------------------------------- 89

FLP-20 ------------------------------- 95

FLP-21 ------------------------------- 66

FLP-22 ------------------------------- 93

FLP-23 ------------------------------- 83

FLP-24 ------------------------------- 69

FLP-25 ------------------------------- 96

FLP-26 ------------------------------- 84

FLP-27 ------------------------------- 89

FLP-28 ------------------------------- 71

FLP-32 ------------------------------- 81

FLP-33 ------------------------------- 86

FLP-34 ------------------------------- 105

FLP-34’ ------------------------------- 105
