# Supplementary material for: Comparative and Evolutionary Aspects of Gonadotropin-Inhibitory Hormone and FMRFamide-Like Peptide Systems
Source: Front Neurosci. 2018 Oct 18;12:747. doi: 10.3389/fnins.2018.00747 (PMC6200920; doi:10.3389/fnins.2018.00747)
Supplement: Supplementary file 2 [file Table_2.DOCX]

**SUPPLEMENTARY FIGURE 2 | A multiple sequence alignment of human, quail, newt, coelacanth, zebrafish, gar, lamprey, amphioxus GnIH, human, quail, turtle, zebrafish, gar, lamprey, amphioxus NPFF, fruit fly FMRFamide and *C. elegans* FMRFamide-like peptide (FLP) precursors.** Human, quail, newt, coelacanth, zebrafish, gar, lamprey, amphioxus GnIH, vertebrate NPFF, fruit fly FMRFamide and *C. elegans* FLP precursor polypeptides were aligned by CLUSTALW Multiple Sequence Alignment software. Multiple alignment parameters were as follows: Gap open penalty 10, Gap extension penalty 0.05, Hydrophilic residues GPSNDQERK, Weight matrix GONNET. Accession numbers are human (*Homo sapiens*) GnIH precursor (Human_GnIH; NP_071433.3), Japanese quail (*Coturnix japonica*) GnIH precursor (Quail_GnIH; XP_015709159.1), Japanese fire belly newt (*Cynops pyrrhogaster*) GnIH precursor (Newt_LPXRFamide; BAJ78290.1), West Indian Ocean coelacanth (*Latimeria chalumnae*) GnIH precursor (Coelacanth_LPXRFamide; XP_005993154.1), zebrafish (*Danio rerio*) GnIH precursor (Zebrafish_LPXRFamide, NP_001076418.1), spotted gar (*Lepisosteus oculatus*) GnIH precursor (Gar_LPXRFamide; XP_015213317.1), sea lamprey (*Petromyzon marinus*) GnIH precursor (Lamprey_LPXRFamide; BAL52329.1), Japanese amphioxus (*Branchiostoma japonicum*) GnIH precursor (B._japonicum_RFamide; BAO77760.1), human NPFF precursor isoform 1 (Human_NPFF_isoform_1; NP_003708.1), human NPFF precursor isoform 2 (Human_NPFF_isoform_2; NP_001307225.1), Japanese quail NPFF precursor (Quail_NPFF; XP_015705838.1), Western painted turtle (*Chrysemys picta bellii*) NPFF precursor (Turtle_NPFF; XP_005307776.1), zebrafish NPFF precursor (Zebrafish_NPFF; BAF34891.1), spotted gar NPFF precursor isoform X2 (Gar_NPFF_isoform_2; XP_015199730.1), sea lamprey NPFF precursor (Lamprey_PQRFamide; BAE79779.1), Florida lancelet (*Branchiostoma floridae*) RFamide precursor 1 (B._floridae_RFamide_1; XP_002599251.1), Florida lancelet RFamide precursor 2 (B._floridae_RFamide_2; XP_002609543.1), Fruit fly (*Drosophila melanogaster*) FMRFamide precursor (Fruit_fly_FMRFamide; NP_523669.2), *C. elegans* (*Caenorhabditis elegans*) FLP-1 precursor (AAC46464.1), FLP-2 precursor (NP_001024945.1), FLP-3 precursor (AAC08940.1), FLP-4 precursor (AAC08941.1), FLP-5 precursor (AAC08942.1), FLP-6 precursor (AAC08943.1), FLP-7 precursor (AAC08944.1), FLP-8 precursor (AAC08945.1), FLP-9 precursor (AAC08946.1), FLP-10 precursor (AAC08947.1), FLP-11 precursor (NP_001024752.1), FLP-12 precursor (AAC08950.1), FLP-13 precursor (AAC08951.1), FLP-14 precursor (NP_499682.2), FLP-15 precursor (NP_499820.1), FLP-16 precursor (NP_001022091.1), FLP-17 precursor (NP_503051.1), FLP-18 precursor (NP_508514.2), FLP-19 precursor (NP_509776.1), FLP-20 precursor (NP_509574.2), FLP-21 precursor (NP_505011.2), FLP-22 precursor (NP_492344.2), FLP-23 precursor (AAY18633.1), FLP-24 precursor (AAW78866.1), FLP-25 precursor (NP_001022665.1), FLP-26 precursor (NP_741827.1), FLP-27 precursor (NP_495111.1), FLP-28 precursor (NP_001024947.1), FLP-32 precursor (NP_510551.2), FLP-33 precursor (NP_871818.1), FLP-34 precursor isoform 1 (FLP-34; NP_001300170.1), FLP-34 precursor isoform 2 (FLP-34’; NP_503365.1).

**Supplementary Figure 2-1**

FLP-7 --MLGSRFLLLALGLLVLVLAEESAEQQVQEPTELEKSGEQLSEEDLIDE

FLP-16 --MNFSGFEFSSIVAFFLLILQLSTAAVL--PADYAYG--------VADE

FLP-34 -----MHSLITELLIFFTVLLSVSVLSLP------------------LEK

FLP-34’ -----MQFQFLMALIFVALVLTDSVLSLP------------------LEK

Human_GnIH -----MEIISSKLFILLTLATSSLLTSNIFCADELVMSN---LHSKEN-Y

Quail_GnIH -----MEIISTQKFILLTLATVAFLTPHGACLDELMKSS---LESREDDD

Newt_LPXRFamide -----MEILLMSRLLLLTLATLGLASQILGLEDPGRSH----LYGEEGDE

Coelacanth_LPXRFamide -------------MPISEPPTIEVVTKV----------------------

Gar_LPXRFamide ------MTPNSCWPVLLLLGCSVLQTPAAQSADERPLS--------ADQD

Human_NPFF_isoform_1 -----MDSRQAAALLVLL-LLIDGGCAEG--------------------P

Human_NPFF_isoform_2 -----MVPQPPTTCPWKP-VPSPCDLRVQGIC-----------------P

Turtle_NPFF -----MDTRLALLLALLSGTVTTGQCLEGGSVSKETLV---------DEP

Gar_NPFF_isoform_2 -----MDTAAWVTLLGLVLAAAGVGGALKE-EGLESAE---------NLQ

Zebrafish_NPFF -----MDAGVWFMLVGVLLVMADQSRSITQDEALEQNK---------RLL

Quail_NPFF -----MAARAVLALLLLAGAARTGRSCP----------------------

Lamprey_PQRFamide -----MEAKAVSAMLLLALANCVLVSAARGSFSSMEEA---------AMP

Zebrafish_LPXRFamide -----MSYFALLSLALGILSSFMLSEVTALRLPLSGER---------DLN

Fruit_fly_FMRFamide -----MGIALMFLLALYQMQSAIHSEIIDTPNYAGNSLQDADSEVSPPQD

FLP-5 -----MRSVPAFQLPRQHPPFTKQSFLATMSSRSTTIA--------FLFI

FLP-4 -----MNAFSSSLKTFIFSLLFATLLALT---------------------

FLP-1 -----MTLLYQVGLLLLVAATYKVSAECCTPGATSDFCT-----VFSMLS

Lamprey_LPXRFamide ----MLAGFLLLHCLHFAFVAPYPESAHGSPLENQALQY------LTEDD

FLP-21 -----MRLFILLSCLLAWVLAAPYIDQED---------------------

FLP-10 -------MQLSIVFVFFVLCLAAVFAVP----------------------

FLP-2 -----MQVSGILSALFLVLLAVIVSPFQFVQPKRILPIP-----------

FLP-27 ----MFSLTQILTFLLVAITLMTFSSAQP---------------------

FLP-28 ----MFSVRSIFAIFCVLILALSTINAAP---------------------

FLP-17 ----MLSKLVLTTCLLLTISGSSQAASMEEIQSE-------------KFC

FLP-33 -----MRFLILIVAIVLLSAVHGFSVEPR--------------------L

FLP-8 -----MLSGVLFSIFVLAISANASCDVSALTTENEKELG-----LRICHL

FLP-14 ----MMICLPTALLLSAFVVAASGQEAPAGAGASGAAQAPHNPKDCQAIL

FLP-6 -----MNSRGLILTLGVVIAVAFAQQDSEVEREMMKRKS-------AYMR

FLP-11 -----MTQFSALALLLIVFVAASFAQSYDDVSAEKRAMR---------NA

FLP-13 ----MMTSLLTISMFVVAIQAFDSSEIRMLDEQYDTKNPFFQFLENSKRS

FLP-22 -----MNRSMIALCVVLMVSLVSAQVFDLD--------------------

FLP-26 -----MKVMFMLALLFSSLVATSAFRLPF---------------------

FLP-3 ----MISPNHLILLFCVNCAFLVASDATPKRSPLGTMRFGKRAIADEMTF

B._floridae_RFamide_2 MRTLGLLAVGVVLMTFSGVPLIGAQTAYPDSSELGDQRQ----VIIRTRR

FLP-23 -----MLLPKISILLYILVVLQETAAVRG---------------------

FLP-20 -MLGYTQSRVVITLLLFSVFLAVCMATPSG------------------YP

FLP-15 -----MQFSTLIRVAVFAVLAIATLADYD---------------------

FLP-9 --MCVYVCAQTPPIRVLSILSQDSAPIKAHFFFWSRFQR--------KTQ

FLP-18 ----MQRWSGVLLISLCCLLRGALAYTEPIYEIVEEDIPAEDIEVTRTNE

FLP-24 ----MLSSRTSSIILILAILVAIM--------------------------

FLP-32 ------MLSFVQTLILALLCSIVFVEAMP---------------------

FLP-19 -----MSFQLTLFSMLFLLIAVVVGQPIQ---------------------

FLP-25 -----MSHNSMIYLLVAFLVLLCATTEAKKECS---------------ID

FLP-12 -----MNVQVIIALLFCLIATCATQKVKG---------------------

B._japonicum_RFamide --MRTLVVLTWISTLFPFLLAATATGSDPRTTYKVSR---------WDEA

B._floridae_RFamide_1 -----MKLSTALVAVLSCLVYLDGAESIPVRRSQAKPMT---------AV

**Supplementary Figure 2-2**

FLP-7 QKRTPMQRSSMVRFGRSPMQ------------------------------

FLP-16 MSALPDS-GSLFAEQRPSKR------------------------------

FLP-34 KADISTFASAINNAGR----------------------------------

FLP-34’ KADISTFASAINNAGR----------------------------------

Human_GnIH DKYSEPRG--YPKGERS---------------------------------

Quail_GnIH DKYYEIKDSILEEKQRS---------------------------------

Newt_LPXRFamide EDFSESNEDIFEETQRS---------------------------------

Coelacanth_LPXRFamide --FQEASQESLEERQRS---------------------------------

Gar_LPXRFamide RLDPDATHENMQEELRS---------------------------------

Human_NPFF_isoform_1 GGQQE---DQLSAEEDS---------------------------------

Human_NPFF_isoform_2 SSFPD---TPLAQEEDS---------------------------------

Turtle_NPFF DSYLERLSDLLQESADR---------------------------------

Gar_NPFF_isoform_2 RQEGQSLTARLAGVLDG---------------------------------

Zebrafish_NPFF EEDEE--QDNMLERPGA---------------------------------

Quail_NPFF ------------DPIRS---------------------------------

Lamprey_PQRFamide DSDSSLTKDYLAESVHE---------------------------------

Zebrafish_LPXRFamide GFTWGQFSENAQEIPRSLEI------------------------------

Fruit_fly_FMRFamide NDLVDALLGNDQTERAELEFRHPISVIGIDYSKNAVVLHFQKHGRKPRYK

FLP-5 ATLLVFQCVSAQSSAED---------------------------------

FLP-4 AAHPPSSGEEIAEQEEK---------------------------------

FLP-1 TMEQNEVMNFIGENCDG---------------------------------

Lamprey_LPXRFamide ALLIRKLQSRMNTALEG---------------------------------

FLP-21 --ALRVLNAYLEQFGPG---------------------------------

FLP-10 ----------ISDASRA---------------------------------

FLP-2 TSRDQLLRGQLAYLKGT---------------------------------

FLP-27 ---IDEERPIFMERREA---------------------------------

FLP-28 -------NRVLMRFGKR---------------------------------

FLP-17 EKFPTLHMCRLKEELTG---------------------------------

FLP-33 AAFADGGAAELAQEARQ---------------------------------

FLP-8 EAEMQVVQRALQEVMQQT--------------------------------

FLP-14 ANNGDQQEALLCQLSESS--------------------------------

FLP-6 FGRSDGGNPMEMEKRKS---------------------------------

FLP-11 LVRFGRASGGMRNALVR---------------------------------

FLP-13 DRPTRAMDSPLIRFGKR---------------------------------

FLP-22 -------GQQLAGLEQN---------------------------------

FLP-26 -QFFGANEDFNSGLTKR---------------------------------

FLP-3 EEDGYYPSNVMWKRSTVD--------------------------------

B._floridae_RFamide_2 AADTGDVSEVIGPNGQRWFIS-----------------------------

FLP-23 ---------ALFRSGRA---------------------------------

FLP-20 GQELQNVSDDYPIYEEE---------------------------------

FLP-15 -------DNSVGTIPVA---------------------------------

FLP-9 QHRLKKGETFFVSKKKKMN-------------------------------

FLP-18 KQDGRVFSKRDFDGAMPG--------------------------------

FLP-24 ---------AVAQCRN----------------------------------

FLP-32 --SMRPAKKAMRNSLVR---------------------------------

FLP-19 SQNGDLKMQAVQDNSPLN--------------------------------

FLP-25 CQEDGSAAVDLGLVLPP---------------------------------

FLP-12 --SPEVLPAAMYDGELS---------------------------------

B._japonicum_RFamide WRPQRFGRSGRGDHTKDG--------------------------------

B._floridae_RFamide_1 NWNEAFEPLRFGRRSPPS--------------------------------

**Supplementary Figure 2-3**

FLP-7 ------------RSSMVRFGKRSPM---------------QRSSMVRFGK

FLP-16 ------------AQTFVRFGKR-------------------AQTFVRFGK

FLP-34 ----------------LRYGKR--------------------SDPAMWEE

FLP-34’ ----------------LRYGKR--------------------SDPAMWEE

Human_GnIH ----------------LNFEELKDWGPKNV----------IKMSTPAVNK

Quail_GnIH ----------------LNFEEMKDWGSKNF----------MKVNTPTVNK

Newt_LPXRFamide ----------------ANSGEEKEVGVRNM----------VKMSAPLVHR

Coelacanth_LPXRFamide ----------------LSNEQLKEWEPKAT----------IKMKTPIISK

Gar_LPXRFamide ----------------IELERIQDILPSVT----------RKIDVPTIQK

Human_NPFF_isoform_1 ----------------EPLP---------------------PQDAQTSGS

Human_NPFF_isoform_2 ----------------EPLP---------------------PQDAQTSGS

Turtle_NPFF ----------------APRP---------------------LSDERPPGT

Gar_NPFF_isoform_2 ----------------EDAGV--------------------RTDERLLSA

Zebrafish_NPFF ----------------AQMNG--------------------LLEDRLLVE

Quail_NPFF ----------------DPLPVGPGS---------------VAPSGP---S

Lamprey_PQRFamide ----------------DPYRDSFDR---------------ASPDAAGSSS

Zebrafish_LPXRFamide ------------QDFTLNVAPTSGGASSPT----------ILRLHPIIPK

Fruit_fly_FMRFamide YDPELEAKRRSVQDNFMHFGKRQAEQLPPEGSYAESDELEGMAKRAAMDR

FLP-5 -----------------------------------------ADYLEKYQR

FLP-4 -----------------NIASP-------------------DELIPEIVE

FLP-1 ----------DAEVALQKMEKRKPNFMRYG-----------RSAAVKSLG

Lamprey_LPXRFamide ----------------AMVEEVEDCG-------------VAATDEVQGSR

FLP-21 --------------------------------------------------

FLP-10 ------------------------------------------RRQVASEK

FLP-2 -----------------------------------------TVAQPAVND

FLP-27 -----------------------------------------SAFGDIIGE

FLP-28 -----------------------------------------------GGN

FLP-17 --------------SLVELQYLLQDG--------------INNQQQAGAQ

FLP-33 -------------------------------------------ARNAELE

FLP-8 ----------------------------------------DVTLYDQEVP

FLP-14 ----------------MLLAQLG---------------ALVSEGVERLVQ

FLP-6 --------------AYMRFGKRSSGGD-------------EQELVGGDDI

FLP-11 ------------------------------------------FGKRSPLD

FLP-13 ----------------------------------------AADGAPLIRF

FLP-22 ------------------------------------------DARLMEQQ

FLP-26 -----------------------------------------NYYESKPYK

FLP-3 ----------------SSEPVIRDQR---------------TPLGTMRFG

B._floridae_RFamide_2 ----------RDRQRMPRFGKRSVPSVRG-----------YNPAEPALRF

FLP-23 -----------------------------------------------VPF

FLP-20 ----------------------------------------GLQLSAEGTD

FLP-15 -----------------------------------------VDLDYFSNY

FLP-9 ---------------------------------QFYALFLVACIAAMANA

FLP-18 ---------------VLRFGKRGGVWEKRESSVQKKEMPGVLRFGKRAYF

FLP-24 ----------------------------------------------IQYD

FLP-32 -------------------------------------------FGKRADP

FLP-19 -----------------------------------------MEAFNDDSA

FLP-25 -----------------------------------------ELYESTRLS

FLP-12 --------------------------------------------HESVNK

B._japonicum_RFamide -------------WRPQRFGRGRDQGWRPQ---------RFGRTEAGLRE

B._floridae_RFamide_1 ----------------------------------------SLSEDDSDSD

**Supplementary Figure 2-4**

FLP-7 RSPMQRSSMVRFGK---------------------RSPMERSAMVRFGRS

FLP-16 RG----QTFVRFGR---------------------SAPFEQ---------

FLP-34 NNVIIPSSEDQYLY---------------------SEGRYPYALIKR---

FLP-34’ NNVIIPSSEDQYLY---------------------SEGRYPYALIKR---

Human_GnIH MPHSFANLPLRFGRNVQE-------------ERSAGATANLPLRSGRNME

Quail_GnIH VPNSVANLPLRFGRSNPE-------------ERSIKPSAYLPLRFGRAFG

Newt_LPXRFamide MPHASANLPLRFGRAFLE-------------EAKSSPAFYSPLRYESAFD

Coelacanth_LPXRFamide FSNSVINLPLRFGRAFPD-------------GRLSQSLANLPLRLGRALE

Gar_LPXRFamide LYHSVTNLPLRFGR-------------------ASQPVANLPLRFGRGLT

Human_NPFF_isoform_1 LLHYLLQAMERPGR--------------------SQAFLFQPQRFGRNTQ

Human_NPFF_isoform_2 LLHYLLQAMERPGR--------------------SQAFLFQPQRFGRNTQ

Turtle_NPFF LLRSLLYTLQRPGR--------------------SPSFLFQPQRFGRETR

Gar_NPFF_isoform_2 LLRPLLHASQRYGR--------------------SPSFLFQPQRFGRETR

Zebrafish_NPFF MLRSLLHGSQRYER--------------------NPSVLHQPQRFGRGAR

Quail_NPFF ALSALLRSMGRPHS---------------------AATELQPQRFPRGPG

Lamprey_PQRFamide SEQLLLSRLARAFMHFPQRF-----------GRAGPSSLFQPQRFGRGSN

Zebrafish_LPXRFamide PAHLHANLPLRFGRDAQPGTG----------DRAPKSTINLPQRFGRSCT

Fruit_fly_FMRFamide YGRDPKQDFMRFGRDPKQDFMRFGRDPKQDFMRFGRDPKQDFMRFGRDPK

FLP-5 IARAPKPKFIRFGR-----------------------AGAKFIRFGRSRN

FLP-4 QQNFWPPVHLRGLR--------------------SSNGKPTFIRFGKRAS

FLP-1 KKAGSDPNFLRFGRSQPNFLRFG-----------KASGDPNFLRFGRSDP

Lamprey_LPXRFamide ENRAALRSGVGQGR--------------------SSKTLFQPQRFGRGVP

FLP-21 SDRVYYVAEDDHGS------------------------------MKRGLG

FLP-10 RQPKARSGYIRFGK----------------------------RRVDPNAE

FLP-2 NTLGIFEASAMAKR-----------------------LRGEPIRFGKRSP

FLP-27 LKGKGLGGRMRFGKR-------------------SSSPDISLAEMRAIYG

FLP-28 SEGHLGYRFVPAGA----------------------PAIAEYIDVDDVIG

FLP-17 EVQKRKSAFVRFGK--------------------RSAPEEEAMEMEKRKS

FLP-33 FIKRFLPAKERRAP---------------------LEGFEDMSGFLRTID

FLP-8 VMNKRKNEFIRFGKRSDG----------------MEKRKNEFIRFGKRKN

FLP-14 THGLALEEETNEGDND------------------MEKRKHEYLRFGKRKH

FLP-6 DMEKRKSAYMRFGKRSGPQED----------DMPMEKRKSAYMRFGKRSS

FLP-11 EEDFAPESPLQGKR---------------------NGAPQPFVRFGRSGQ

FLP-13 GRAPEASPFIRFGKR--------------------AADGAPLIRFGRAPE

FLP-22 VKRSPSAKWMRFGK---------------------RSPSAKWMRFGKRSP

FLP-26 REFNADDLTLRFGK---------------------RGGAGEPLAFSPDML

FLP-3 KRSAEPFGTMRFGKRNPE-----------------NDTPFGTMRFGKRAS

B._floridae_RFamide_2 GRTSPYQPNLRFGKKLDPARPALRFG-------KKFDPAQPTLRFGKSLD

FLP-23 ERVVGQQDFLRFGR----------------------AGMASGVGGGSEGG

FLP-20 EPHEEKRAVFRMGK-------------------------RAMMRFGKR--

FLP-15 VKKGGPQGPLRFGK---------------------RRGPSGPLRFGKRSS

FLP-9 YEEPDLDALAEFCGKESN-----------------RKYCDQIAQLATQHA

FLP-18 DEKKSVPGVLRFGKRSYFD---------------EKKSVPGVLRFGKRDV

FLP-24 VEEMTPEAAFRYAQ----------------------WGEIPHKRVPSAG-

FLP-32 VGTDDVFLGESYGS--------------------ADPYEYVPERMSNRGP

FLP-19 LYDYLEQSDPSLKS--------------------MEKRWANQVRFGKRAS

FLP-25 NLLARPSSQFKMKR------------------------DYDFVRFGRAAP

FLP-12 ISAQLLNALSELEA---------------------LQEGNQQLKMAEKRR

B._japonicum_RFamide VLGGEAFPLLQMTR-----------------TDLHDDLPAMAVRYTPPAA

B._floridae_RFamide_1 LWPDYTAPQPRFTLP-------------------PQREPMARMRLPNLRE

**Supplementary Figure 2-5**

FLP-7 PMDRSK---MVRFGRSSIDRASMVRLGKRTPMQRSSMVRFGKRSMEFEMQ

FLP-16 --------------------------------------------------

FLP-34 ----------------ALNRDSLVAS-----LNNAERLRFGRK-------

FLP-34’ ----------------ALNRDSLVAS-----LNNAERLRFGRK-------

Human_GnIH V---SL---VRRVPNLPQRFGRTTTAKSVCRMLSDLCQGSMHSPCANDLF

Quail_GnIH E---SL---SRRAPNLSNRSGRSPLARSSIQSLLNLPQR-----FGKSVP

Newt_LPXRFamide E---RI---RKSVPNLPQRFGRYLASKRSIQPLANLPQRFGRAPSAGQFI

Coelacanth_LPXRFamide N---RI---PMAIPNLPQRFGRSPLVKSFMQPLANLPQRFGRSPFYDKFI

Gar_LPXRFamide EGSARK---AKAALNLPQRFGRAPAR---LPPLPAVPQRAVYAPVEEDEK

Human_NPFF_isoform_1 G----------SWRNE-WLSPRAGEGLNSQFWSLAAPQRFGKK-------

Human_NPFF_isoform_2 G----------SWRNE-WLSPRAGEGLNSQFWSLAAPQRFGKK-------

Turtle_NPFF G----------SWGGEGRLSQRGWDSMASQFWSMAVPQRFGKKK------

Gar_NPFF_isoform_2 G----------GLGFEGRIQSRDWETMPPQFWSMAVPQRFGKKK------

Zebrafish_NPFF S----------GLSTEERIQSRDWETVPGQIWSMAVPQRFGKK-------

Quail_NPFF S--------------PSWLSPRSWDPPSAPFWTMATPQRFGRRR------

Lamprey_PQRFamide DD---------EEVPPSLFYRRSWGAPAEKFWMRAMPQRFGRKK------

Zebrafish_LPXRFamide MCARSG---TGPSATLPQRFGRRNIFALDPLRALALYTRTPESPSFPKER

Fruit_fly_FMRFamide QDFMRF---GRTPAEDFMRFGRTPAEDFMRFGRSDNFMRFGRSPHEELRS

FLP-5 T---------------WEDGYASPSVNELYVKRGAKFIRFG---------

FLP-4 P----------------------------------SFIRFGK--------

FLP-1 N-----------FLRFGKAAADPNFLRFGKRSADPNFLRFGRSFDNFDRE

Lamprey_LPXRFamide PPAADCPESAAASWAGLQDGNADRASRSEPFWHRTRPQRFGKRGGDPASP

FLP-21 P----------------------------------RPLRFG---------

FLP-10 L-----------------------------LYLDQLLI------------

FLP-2 R----------------------------------EPIRFGKRFNPLPDY

FLP-27 G-------------------------------DQSNIFNFK---------

FLP-28 G-----------------------------------DDRF----------

FLP-17 A--------------FVRFGRSFGMEPQITEKRKSQYIRFGK--------

FLP-33 G---------------------------------IQKPRFG---------

FLP-8 E--------------FIRFGRSDKGLGLDDNDVSSEFFGYTSDVFYL---

FLP-14 EY---------------LRFGKRKHEYLRFGKRKHEYLRFGRK-------

FLP-6 D-------------MEVIGNEGVDGDAHDLFKRKSAYMRFGKRSMGEEED

FLP-11 LD------------------------HMHDLLSTLQKLKFANNK------

FLP-13 ASP--------FIRFGKRASPSAPLIRFGRSPSAVPLIRFGRSAAAPLIR

FLP-22 S---------------------------------AKWMRFGKRSGAEAVS

FLP-26 S------------------------------------LRFGK--------

FLP-3 EDALFG-TMRFGKREDGNAPFGTMKFGKREAEEPLGTMRFGKRSADDSAP

B._floridae_RFamide_2 PNEPSLR-----FGRDINEPAQEILRAEKSFDPSEPSLRFGKKYDPFEPT

FLP-23 P-----------------------------DDVKNSYIRVNGEPEIVYQ-

FLP-20 -----------------------------------AMMRFGKRSVFRLG-

FLP-15 F---------------------------HVAPAAEDVASWYQ--------

FLP-9 IG--------------------INQEQVRMEKRKPSFVRFGKRSGYPLVI

FLP-18 PMDKRE-----IPGVLRFGKRDYMADSFDKRSEVPGVLRFGKRDVPGVLR

FLP-24 ----------------------------------DMMVRFGKRSI-----

FLP-32 S----------------------------------SVLLY----------

FLP-19 W---------------------------------ASSVRFG---------

FLP-25 IK-----------------------------KASYDYIRFGRK-------

FLP-12 N--------------------------------KFEFIRFGRK-------

B._japonicum_RFamide RLRALPLLRLYDRGALSQLINGPPKQPATNREVYPPSLRMIRAAAEGLRG

B._floridae_RFamide_1 D--------------------------PTDQLTRSAVLRLVADLIQARQQ

**Supplementary Figure 2-6**

FLP-7 SNEKNIEDSE----------------------------------------

FLP-16 --------------------------------------------------

FLP-34 --------------------------------------------------

FLP-34’ --------------------------------------------------

Human_GnIH YSMTCQHQEIQNPDQKQSRRLLFKKID-----------------------

Quail_GnIH ISLSQGVQESE-PGM-----------------------------------

Newt_LPXRFamide QTLANLPQRFGRSIDLHKLCNFANTYAKGGQESGYGDKRMLDADNGPEEE

Coelacanth_LPXRFamide QSVANLPQRFGRSPSVSNYPHSTVAFP-----------------------

Gar_LPXRFamide SSQEL---------------------------------------------

Human_NPFF_isoform_1 --------------------------------------------------

Human_NPFF_isoform_2 --------------------------------------------------

Turtle_NPFF --------------------------------------------------

Gar_NPFF_isoform_2 --------------------------------------------------

Zebrafish_NPFF --------------------------------------------------

Quail_NPFF --------------------------------------------------

Lamprey_PQRFamide --------------------------------------------------

Zebrafish_LPXRFamide TQVHDYMFETVEDSEETVKNTDYTALD-----------------------

Fruit_fly_FMRFamide PKQDFMRFGRPDNFMRFGRSAPQDFVRSGKMDSNFIRFGKSLKPAAPESK

FLP-5 --------------------------------------------------

FLP-4 --------------------------------------------------

FLP-1 SRKPNFLRFGK---------------------------------------

Lamprey_LPXRFamide M-------------------------------------------------

FLP-21 --------------------------------------------------

FLP-10 --------------------------------------------------

FLP-2 DFQ-----------------------------------------------

FLP-27 --------------------------------------------------

FLP-28 --------------------------------------------------

FLP-17 --------------------------------------------------

FLP-33 --------------------------------------------------

FLP-8 --------------------------------------------------

FLP-14 --------------------------------------------------

FLP-6 HDMMKRKSAYMRFGR-----------------------------------

FLP-11 --------------------------------------------------

FLP-13 FGRASSAPLIRFGRK-----------------------------------

FLP-22 EQDY----------------------------------------------

FLP-26 --------------------------------------------------

FLP-3 FGTMRFGKRNPLGTMRFGK-------------------------------

B._floridae_RFamide_2 LRFGRQVDPSAPAYRFGRHNPAKPSLRFGRDAASEQLDSSED--------

FLP-23 --------------------------------------------------

FLP-20 --------------------------------------------------

FLP-15 --------------------------------------------------

FLP-9 DDEEMRMDKRKPSFVRFGRK------------------------------

FLP-18 FGKRSDLEEHYAGVLLKKSVPGVLRFGRK---------------------

FLP-24 --------------------------------------------------

FLP-32 --------------------------------------------------

FLP-19 --------------------------------------------------

FLP-25 --------------------------------------------------

FLP-12 --------------------------------------------------

B._japonicum_RFamide FAHQQDKDTGESFAPPRSNDDWLAEIQRLGLRGRKRRDVS----------

B._floridae_RFamide_1 DLLDN---------------------------------------------

**Supplementary Figure 2-7**

FLP-7 --------------------------------------------------

FLP-16 --------------------------------------------------

FLP-34 --------------------------------------------------

FLP-34’ --------------------------------------------------

Human_GnIH -------DAELKQEK-----------------------------------

Quail_GnIH --------------------------------------------------

Newt_LPXRFamide QKEEGATQNNWNQNHNQMVM------------------------------

Coelacanth_LPXRFamide ------VQFERYQQTN----------------------------------

Gar_LPXRFamide --------------------------------------------------

Human_NPFF_isoform_1 --------------------------------------------------

Human_NPFF_isoform_2 --------------------------------------------------

Turtle_NPFF --------------------------------------------------

Gar_NPFF_isoform_2 --------------------------------------------------

Zebrafish_NPFF --------------------------------------------------

Quail_NPFF --------------------------------------------------

Lamprey_PQRFamide --------------------------------------------------

Zebrafish_LPXRFamide --------------------------------------------------

Fruit_fly_FMRFamide PVKSNQGNPGERSPVDKAMTELFKKQELQDQQVKNGAQATTTQDGSVEQD

FLP-5 --------------------------------------------------

FLP-4 --------------------------------------------------

FLP-1 --------------------------------------------------

Lamprey_LPXRFamide --------------------------------------------------

FLP-21 --------------------------------------------------

FLP-10 --------------------------------------------------

FLP-2 --------------------------------------------------

FLP-27 --------------------------------------------------

FLP-28 --------------------------------------------------

FLP-17 --------------------------------------------------

FLP-33 --------------------------------------------------

FLP-8 --------------------------------------------------

FLP-14 --------------------------------------------------

FLP-6 --------------------------------------------------

FLP-11 --------------------------------------------------

FLP-13 --------------------------------------------------

FLP-22 --------------------------------------------------

FLP-26 --------------------------------------------------

FLP-3 --------------------------------------------------

B._floridae_RFamide_2 --------------------------------------------------

FLP-23 --------------------------------------------------

FLP-20 --------------------------------------------------

FLP-15 --------------------------------------------------

FLP-9 --------------------------------------------------

FLP-18 --------------------------------------------------

FLP-24 --------------------------------------------------

FLP-32 --------------------------------------------------

FLP-19 --------------------------------------------------

FLP-25 --------------------------------------------------

FLP-12 --------------------------------------------------

B._japonicum_RFamide --------------------------------------------------

B._floridae_RFamide_1 --------------------------------------------------

**Supplementary Figure 2-8**

FLP-7 -----

FLP-16 -----

FLP-34 -----

FLP-34’ -----

Human_GnIH -----

Quail_GnIH -----

Newt_LPXRFamide -----

Coelacanth_LPXRFamide -----

Gar_LPXRFamide_peptide -----

Human_NPFF_isoform_1 -----

Human_NPFF_isoform_2 -----

Turtle_NPFF -----

Gar_NPFF_isoform_2 -----

Zebrafish_NPFF -----

Quail_NPFF -----

Lamprey_PQRFamide -----

Zebrafish_LPXRFamide -----

Fruit_fly_FMRFamide QFFGQ

FLP-5 -----

FLP-4 -----

FLP-1 -----

Lamprey_LPXRFamide -----

FLP-21 -----

FLP-10 -----

FLP-2 -----

FLP-27 -----

FLP-28 -----

FLP-17 -----

FLP-33 -----

FLP-8 -----

FLP-14 -----

FLP-6 -----

FLP-11 -----

FLP-13 -----

FLP-22 -----

FLP-26 -----

FLP-3 -----

B._floridae_RFamide_2 -----

FLP-23 -----

FLP-20 -----

FLP-15 -----

FLP-9 -----

FLP-18 -----

FLP-24 -----

FLP-32 -----

FLP-19 -----

FLP-25 -----

FLP-12 -----

B._japonicum_RFamide -----

B._floridae_RFamide_1 -----
