# Supplementary material for: Comparative and Evolutionary Aspects of Gonadotropin-Inhibitory Hormone and FMRFamide-Like Peptide Systems
Source: Front Neurosci. 2018 Oct 18;12:747. doi: 10.3389/fnins.2018.00747 (PMC6200920; doi:10.3389/fnins.2018.00747)
Supplement: Supplementary file 3 [file Table_3.DOCX]

**SUPPLEMENTARY FIGURE 3 | A multiple sequence alignment of human GnIH receptors (GPR147, GPR74) and *C. elegans* FMRFamide-like peptide (FLP) receptors.** Human GnIH receptors (GPR147, GPR74) and *C. elegans* FLP receptors were aligned by CLUSTALW Multiple Sequence Alignment software. Multiple alignment parameters were as follows: Gap open penalty 10, Gap extension penalty 0.05, Hydrophilic residues GPSNDQERK, Weight matrix GONNET. The accession numbers of human GnIH receptors are GPR147 (NPFF1; NP_071429.1), GPR74 (NPFF2; AAG41398.1). WormBase IDs of C. elegans FLP receptors are NPR-1 (WP:CE06941), NPR-3 (WP:CE08056), NPR-4 isoform a (WP:CE37317), NPR-4 isoform b (WP:CE50076), NPR-4 isoform c (WP:CE50063), NPR-4 isoform d (WP:CE50035), NPR-5a (WP:CE33345), NPR-5b (WP:CE36962), NPR-10a (WP:CE19767), NPR-10b (WP:CE36989), NPR-11 (WP:CE47199), FRPR-18 isoform a (WP:CE28679), FRPR-18 isoform b (WP:CE29349), FRPR-18 isoform c (WP:CE52203), NPR-22 isoform a (WP:CE31260), NPR-22 isoform b (WP:CE38456), EGL-6a (WP:CE04219), EGL-6b (WP:CE43400). Asterisk (*) indicates positions which have a single, fully conserved residue. Colon (:) indicates conservation between groups of strongly similar properties – scoring >0.5 in the Gonnet PAM 250 matrix. Period (.) indicates conservation between groups of weakly similar properties – scoring 60.5 in the Gonnet PAM 250 matrix.

**Supplementary Figure 3-1**

NPR-22a ----MDEGGGIGSSLLSRITTTASEIMMRNEPTTTENPAVQEMN-HIYHLTPSMKMLCIL

NPR-22b ----MDEGGGIGSSLLSRITTTASEIMMRNEPTTTENPAVQEMN-HIYHLTPSMKMLCIL

GPR147 ----------MEGEPSQPPNSSWP--LSQNGTNTEATPATNLTFSSYYQHTSPVAAMFIV

GPR74 ----------MNEKWDTNSSENWHPIWNVNDTKHHLYSDINITYVNYYLHQPQVAAIFII

NPR-10a --------------------------MSSSNHCIDIRAYLWQT-KHDLTLHPIPIAILAT

NPR-10b --------------------------MSSSNHCIDIRAYLWQT-KHDLTLHPIPIAILAT

NPR-4a --------------------------MNGS-DCLNLNSELWLY-REDLSSRWYIMLVFAF

NPR-4b ----MLLEIGTITIGNITRARKFGERMNGS-DCLNLNSELWLY-REDLSSRWYIMLVFAF

NPR-4c ----MLLEIGTITIGNITRARKFGERMNGS-DCLNLNSELWLY-REDLSSRWYIMLVFAF

NPR-4d --------------------------MNGS-DCLNLNSELWLY-REDLSSRWYIMLVFAF

NPR-5a MVSSAATISTISTTTTPSTISNVITSHSNNGSCIQIAEAIAAQGIDDITVDFYIRSIFTF

NPR-5b MVSSAATISTISTTTTPSTISNVITSHSNNGSCIQIAEAIAAQGIDDITVDFYIRSIFTF

FRPR-18a ---------------------------------------------------MESQQLMAC

FRPR-18c ---------------------------------------------------MESQQLMAC

FRPR-18b ---------------------------------------------------MESQQLMAC

EGL-6a ----------------------MGKWTELGLSGRENATNVSSRAITDKSFLQYYDEIHIP

EGL-6b ---------------------------------MNDTLICTYFECYIHMFAMYYDEIHIP

NPR-3 --------------------------MEGGRNCVMT----VQQWQPEYNDMNQIRAIFSL

NPR-1 -----------------------------MEVENFTDCQVYWKVYPDPSQSIYAIVPFLT

NPR-11 ----------------MGSVNESCDNYVEIFNKINYFFRDDQVINGTEYSPKEFGYFITF

NPR-22a FYSILCVCCVYGNVLVILVIVYFKRLRTATNILILNLAVADLLISVFCIPFSYWQVLIYD

NPR-22b FYSILCVCCVYGNVLVILVIVYFKRLRTATNILILNLAVADLLISVFCIPFSYWQVLIYD

GPR147 AYALIFLLCMVGNTLVCFIVLKNRHMHTVTNMFILNLAVSDLLVGIFCMPTTLVDNLITG

GPR74 SYFLIFFLCMMGNTVVCFIVMRNKHMHTVTNLFILNLAISDLLVGIFCMPITLLDNIIAG

NPR-10a IYTIIVVVGVTGNLLVVMSVMRFKVLQSVRNMFIVSLSVSDIFVAIVSGSVTPITAFSKV

NPR-10b IYTIIVVVGVTGNLLVVMSVMRFKVLQSVRNMFIVSLSVSDIFVAIVSGSVTPITAFSKV

NPR-4a LYLIIIAAGIIGNSCVILAITRNKSLQTVPNLFILSLSCSDIVVCCTSATITPITAFKKE

NPR-4b LYLIIIAAGIIGNSCVILAITRNKSLQTVPNLFILSLSCSDIVVCCTSATITPITAFKKE

NPR-4c LYLIIIAAGIIGNSCVILAITRNKSLQTVPNLFILSLSCSDIVVCCTSATITPITAFKKE

NPR-4d LYLIIIAAGIIGNSCVILAITRNKSLQTVPNLFILSLSCSDIVVCCTSATITPITAFKKE

NPR-5a LYGFLFVLGIFGNGGVLWAVARNKRLQSARNVFLLNLIFTDLILVFTAIPVTPWYAMTKD

NPR-5b LYGFLFVLGIFGNGGVLWAVARNKRLQSARNVFLLNLIFTDLILVFTAIPVTPWYAMTKD

FRPR-18a AILVIVLVGIFGNSLSFILFSRPHMRSSSVNVLLCALSFFDFSLLTLSIPIFVIPNLDLW

FRPR-18c AILVIVLVGIFGNSLSFILFSRPHMRSSSVNVLLCALSFFDFSLLTLSIPIFVIPNLDLW

FRPR-18b AILVIVLVGIFGNSLSFILFSRPHMRSSSVNVLLCALSFFDFSLLTLSIPIFVIPNLDLW

EGL-6a LSISICIFGAASNVFNIIVLTRKRMR-TPINILLTGLSIAQWLLATNYFLYLLLEYYRYQ

EGL-6b LSISICIFGAASNVFNIIVLTRKRMR-TPINILLTGLSIAQWLLATNYFLYLLLEYYRYQ

NPR-3 LYLLVWVGAIVGNTLVLYVLTFNQVSLSVRTVFVGCLAGSDLLMCLFSLPITAISIFSRV

NPR-1 VYLFLFFLGLFGNVTLIYVTCSHKALLSVQNIFILNLAASDCMMCILSLPITPITNVYKN

NPR-11 AYMLIILFGAIGNFLTIIVVILNPAMRTTRNFFILNLALSDFFVCIVTAPTTLYTVLYMF

: .* : ..:: * : :

**Supplementary Figure 3-2**

NPR-22a DQRWLFGSMMCSLLAFLQAMAVFLS---AWTLVVISFDRWMAIMFLLTPNI---RITRRR

NPR-22b DQRWLFGSMMCSLLAFLQAMAVFLS---AWTLVVISFDRWMAIMFLLTPNI---RITRRR

GPR147 ---WPFDNATCKMSGLVQGMSVSAS---VFTLVAIAVERFRCIVHPFRE-----KLTLRK

GPR74 ---WPFGNTMCKISGLVQGISVAAS---VFTLVAIAVDRFQCVVYPFKP-----KLTIKT

NPR-10a ---WLFGGPLCHLLPLLQGTALSFS---TLTLTAIAIDRYILICHPTKEP-----IRKDQ

NPR-10b ---WLFGGPLCHLLPLLQGTALSFS---TLTLTAIAIDRYILICHPTKEP-----IRKDQ

NPR-4a ---WIFGEALCRIAPFIAGISLCFS---TFTLTAISIDRYILIRFPMRKP-----ITHYQ

NPR-4b ---WIFGEALCRIAPFIAGISLCFS---TFTLTAISIDRYILIRFPMRKP-----ITHYQ

NPR-4c ---WIFGEALCRIAPFIAGISLCFS---TFTLTAISIDRYILIRFPMRKP-----ITHYQ

NPR-4d ---WIFGEALCRIAPFIAGISLCFS---TFTLTAISIDRYILIRFPMRKP-----ITHYQ

NPR-5a ---WAFGSVMCHLVPLSNSCSVFVT---SWSLTAISLDKFLHINDPTKQP-----VSIRQ

NPR-5b ---WAFGSVMCHLVPLSNSCSVFVT---SWSLTAISLDKFLHINDPTKQP-----VSIRQ

FRPR-18a ANDLSLSTYMAYILKLIYPINLMMQTCSVYIMVMITLERWVAVCRPLQVR---VWCTPRK

FRPR-18c ANDLSLSTYMAYILKLIYPINLMMQTCSVYIMVMITLERWVAVCRPLQVR---VWCTPRK

FRPR-18b ANDLSLSTYMAYILKLIYPINLMMQTCSVYIMVMITLERWVAVCRPLQVR---VWCTPRK

EGL-6a CVQLLWSEAFTRYRFFNVNLNTVFHTIAFTTTIVVAVFRYCALKFPIQANRFIYKCQPAI

EGL-6b CVQLLWSEAFTRYRFFNVNLNTVFHTIAFTTTIVVAVFRYCALKFPIQANRFIYKCQPAI

NPR-3 ---WVFPAIFCKLIGVFQGGTIFVS---SFTLTVIALDRCVLILRPNQEI-----VNFPR

NPR-1 ---WYFGNLLCHLIPCIQGISIFVC---TFSLGAIALDRYILVVRPHSTP-----LSQRG

NPR-11 ---WPFSRTLCKIAGSLQGFNIFLS---TFSIASIAVDRYVLIIFPTKRE-----RQQNL

::. : :

NPR-22a ALYLVAATWIFSILMALPLLFTTRFFEDQDGLPNCGEN-------WTYFGDS--GEQVRK

NPR-22b ALYLVAATWIFSILMALPLLFTTRFFEDQDGLPNCGEN-------WTYFGDS--GEQVRK

GPR147 ALVTIAVIWALALLIMCPSAVTLTVTREEHH-FMVDARNRSYPLYSCWEAWP--EKGMRR

GPR74 AFVIIMIIWVLAITIMSPSAVMLHVQEEKYYRVRLNSQNKTSPVYWCREDWP--NQEMRK

NPR-10a ALKMISFNSAISVGLSVPLFMKQELMQFRN----------YCGEYCSENWGP--DAYLRS

NPR-10b ALKMISFNSAISVGLSVPLFMKQELMQFRN----------YCGEYCSENWGP--DAYLRS

NPR-4a AVGVIAIICAFAATITSPIMFKQKLGEFEN----------FCGQYCTENWGA--NESQRK

NPR-4b AVGVIAIICAFAATITSPIMFKQKLGEFEN----------FCGQYCTENWGA--NESQRK

NPR-4c AVGVIAIICAFAATITSPIMFKQKLGEFEN----------FCGQYCTENWGA--NESQRK

NPR-4d AVGVIAIICAFAATITSPIMFKQKLGEFEN----------FCGQYCTENWGA--NESQRK

NPR-5a ALAITFLIWIVSTLINLPYLMSFEHVDGSFYVQPGETP--YCGHFCDEANWQ--SENSRK

NPR-5b ALAITFLIWIVSTLINLPYLMSFEHVDGSFYVQPGETP--YCGHFCDEANWQ--SENSRK

FRPR-18a SRNAILVIIVSAFLYNFVRFFEYRFVVTESG--------ALYEKWLRDPGKH--RWYYVG

FRPR-18c SRNAILVIIVSAFLYNFVRFFEYRFVVTESG--------ALYEKWLRDPGKH--RWYYVG

FRPR-18b SRNAILVIIVSAFLYNFVRFFEYRFVVTESG--------ALYEKWLRDPGKH--RWYYVG

EGL-6a AANVIIWIIIPIISLPLFFISEVKIVARDH---------VAYDLQCEMEG----PLYDLS

EGL-6b AANVIIWIIIPIISLPLFFISEVKIVARDH---------VAYDLQCEMEG----PLYDLS

NPR-3 AVFIVFCIWLLGYSLALPVGIYSDIAVYDEICG------TFCEENWPDFNPDTGRSGIRR

NPR-1 AFLTTVLLWILSFVVTLPYAFNMQMIEYTEER--------ICGYFCTEKWES---AKSRR

NPR-11 SFCFFIMIWVISLILAVPLLQASDLTPVFVEPS-----CDLALYICHEQNEIWEKMIISK

:

**Supplementary Figure 3-3**

NPR-22a -VYSSMVLILQYVVPQAVLIITYTHIGIKMWNSRVPGMQNGAT-----------------

NPR-22b -VYSSMVLILQYVVPQAVLIITYTHIGIKMWNSRVPGMQNGAT-----------------

GPR147 -VYTTVLFSHIYLAPLALIVVMYARIARKLCQAPGP-APGGE------------------

GPR74 -IYTTVLFANIYLAPLSLIVIMYGRIGISLFRAAVP-HTGRKN-----------------

NPR-10a -VYGTVVFIIQFVFPLITITFCYASISIKLRRGVFVRGSQKE------------------

NPR-10b -VYGTVVFIIQFVFPLITITFCYASISIKLRRGVFVRGSQKE------------------

NPR-4a -IYGAALMFLQLVIPLTIIIISYTAISLKIGQSMILKGAKKQKTDN--------------

NPR-4b -IYGAALMFLQLVIPLTIIIISYTAISLKIGQSMILKGAKKQKTDN--------------

NPR-4c -IYGAALMFLQLVIPLTIIIISYTAISLKIGQSMILKGAKKQKTDN--------------

NPR-4d -IYGAALMFLQLVIPLTIIIISYTAISLKIGQSMILKGAKKQKTDN--------------

NPR-5a -IYGTTVMLLQFVVPMAVITYCYFKILQKVSKDMIIQNAQFCQS----------------

NPR-5b -IYGTTVMLLQFVVPMAVITYCYFKILQKVSKDMIIQNAQFCQS----------------

FRPR-18a -YYTILYIVTHFLVPFSVMAFANGHVIVAMCKLSKTRQMLTRQQQREQSTTVMLLIVTFV

FRPR-18c -YYTILYIVTHFLVPFSVMAFANGHVIVAMCKLSKTRQMLTRQ-----------------

FRPR-18b -YYTILYIVTHFLVPFSVMAFANGHVIVAMCKLSKTRQMLTRQQQREQSTTVMLLIVTFV

EGL-6a -YQESPLLVSAVFWAFGIVFKLLPSLILSILLIALIRSLKSVE-----------------

EGL-6b -YQESPLLVSAVFWAFGIVFKLLPSLILSILLIALIRSLKSVE-----------------

NPR-3 -AYGLSVLVLQFGIPALISSICYWMISRVMSDQLARRRGHNIR-----------------

NPR-1 -AYTMIVMLAQFVVPFAVMAFCYANIVSVLSKRAQTKIRKMVERTSALESSCAFPSHG--

NPR-11 GTYTLAVLITQYAFPLFSLVFAYSRIAHRMKLRFANRNQNVTTNTN--------------

: . : :

NPR-22a ----------------------KKMIVDRHESVKKLVPMVILISALFALCWLPLLILINV

NPR-22b ----------------------KKMIVDRHESVKKLVPMVILISALFALCWLPLLILINV

GPR147 ----------------------EAADPRASRRRARVVHMLVMVALFFTLSWLPLWALLLL

GPR74 ----------------------QEQWHVVSRKKQKIIKMLLIVALLFILSWLPLWTLMML

NPR-10a --------------------LMSEARRQLTQRRLRTNRMLIIMTVTFALSWLPSVGFNFL

NPR-10b --------------------LMSEARRQLTQRRLRTNRMLIIMTVTFALSWLPSVGFNFL

NPR-4a -----------------WEMELSDQQRIAVKRRQRTNRMLIGMVVAFACSWIWSVTFNIL

NPR-4b -----------------WEMELSDQQRIAVKRRQRTNRMLIGMVVAFACSWIWSVTFNIL

NPR-4c -----------------WEMELSDQQRIAVKRRQRTNRMLIGMVVAFACSWIWSVTFNIL

NPR-4d -----------------WEMELSDQQRIAVKRRQRTNRMLIGMVVAFACSWIWSVTFNIL

NPR-5a --------------------LTQKQRSDATSRKKKVNYILIAMVVTFIGCWLPLTLLNLV

NPR-5b --------------------LTQKQRSDATSRKKKVNYILIAMVVTFIGCWLPLTLLNLV

FRPR-18a FAICNTLPFLLNVSESIFPTLFQDESTRGLAYWLNDLSNLLVVLNSGTTFIIYFTFSEKY

FRPR-18c -------------------------STRGLAYWLNDLSNLLVVLNSGTTFIIYFTFSEKY

FRPR-18b FAICNTLPFLLNVSESIFPTLFQDESTRGLAYWLNDLSNLLVVLNSGTTFIIYFTFSEKY

EGL-6a ---------------------------RRRKNWKRTQG-ANICTNSERKAKRKLTTRPRT

EGL-6b ---------------------------RRRKNWKRTQG-ANICTNSERKAKRKLTTRPRT

NPR-3 ----------------------PESETKLVNRKTRANRMMIVMVVGFVLAWMPFNAVNLY

NPR-1 -------LEQYENELNEFLDKQEKEKQRVVLQNRRTTSILVTMVVWFGITWLPHNVISLI

NPR-11 ---------------------TSQRRRSVVERQRRTHLLLVCVVAVFAVAWLPLNVFHIF

.

**Supplementary Figure 3-4**

NPR-22a IPEFYPD------INSWGYI-LYLWWFAHGLAMSHSMVNPIIYFIRN-ARFREGF-CFFS

NPR-22b IPEFYPD------INSWGYI-LYLWWFAHGLAMSHSMVNPIIYFIRN-ARFREGF-CFFS

GPR147 IDYGQLS------APQLHLVTVYAFPFAHWLAFFNSSANPIIYGYFN-ENFRRGFQAAFR

GPR74 SDYADLS------PNELQIINIYIYPFAHWLAFGNSSVNPIIYGFFN-ENFRRGFQEAFQ

NPR-10a RDYSAL-------PGIIDSQDYLFGIIFHCISMTSVIVNPFLYGYCN-EHFRAAFAALLD

NPR-10b RDYSAL-------PGIIDSQDYLFGIIFHCISMTSVIVNPFLYGYCN-EHFRAAFAALLD

NPR-4a RDYEYL-------PELIKTQEYIFGIATHCIAMTSTVWNPLLYAVLN-LQLRAAFIDLMP

NPR-4b RDYEYL-------PELIKTQEYIFGIATHCIAMTSTVWNPLLYAVLN-LQLRAAFIDLMP

NPR-4c RDYEYL-------PELIKTQEYIFGIATHCIAMTSTVWNPLLYAVLN-LQLRAAFIDLMP

NPR-4d RDYEYL-------PELIKTQEYIFGIATHCIAMTSTVWNPLLYAVLN-LQLRAAFIDLMP

NPR-5a KDFKKE-------PEWLKRQPFFWAINAHVIAMSLVVWNPLLFFWLTRKQKRSGLSKILN

NPR-5b KDFKKE-------PEWLKRQPFFWAINAHVIAMSLVVWNPLLFFWLTRKQKRSGLSKILN

FRPR-18a RQTLVFI------LKNGCCATVSD--YNNYTAMSRTASMRISSETGG--QIQRQGSKMSN

FRPR-18c RQTLVFI------LKNGCCATVSD--YNNYTAMSRTASMRISSETGG--QIQRQGSKMSN

FRPR-18b RQTLVFI------LKNGCCATVSD--YNNYTAMSRTASMRISSETGG--QIQRQGSKMSN

EGL-6a TRMLVII------LLLCVMVELPMGILNLCVAIYGEEFGNRYYDPVG--NLMEMLTLLYS

EGL-6b TRMLVII------LLLCVMVELPMGILNLCVAIYGEEFGNRYYDPVG--NLMEMLTLLYS

NPR-3 RDLFGI-----------SKWYSTVFALCHVCAMCSAVLNPIIYSWFN-PQFRQSITTLFK

NPR-1 IEYDDTQSFFRLYGRDDYDISYLLNLFTHSIAMSNNVLNPVLYAWLNPSFRQLVIKTYFG

NPR-11 NTFELVN-----------SFSVTTFSICHCLAMCSACLNPLIYAFFN-HNFRIEFMHLFD

::

NPR-22a SKLLPCISFKELRLLTDNTSRRHRLRD-----IHEVES--LTGKHVVRHVSSKPDHS---

NPR-22b SKLLPCISFKELRLLTDNTSRSFRNRSRFSGVINPTSSDEKPATSLTRYSRSGVLDR---

GPR147 ARLCPRPSGSHKEAYSERPGGLLHRRVFVVVRPSDSGLPSESGPSSGAPRPGRLPLRN--

GPR74 LQLCQKR-AKPMEAYA------LKAKSHVLINTSN-QLVQES--TFQNPHGETLLYR---

NPR-10a TVKAACGMRRVSPGNPACSQLLSTHFESTTRR----------------------------

NPR-10b TVKAACGMRR---GNPACSQLLSTHFESTTRR----------------------------

NPR-4a HWLR----RHLNLEGDNSSPLLNHPTMTITNKYGSTATKTVKATYINTSNGQPYVSTSLV

NPR-4b HWLR----RHLNLEGDNSSPLLNHPTMTITNKYGSTATKTVKATYINTSNGQPYVSTSLV

NPR-4c HWLR----RHLNLEGDNSSPLLNHPTMTITNK----------------------------

NPR-4d HWLR----RHLNLEGDNSSPLLNHPTMTITNK----------------------------

NPR-5a STEGSKKAGGSGLRGIQLHDLLPTSTHSDR------------------------------

NPR-5b STEIVSSFASRVSNSIRRSTFRRNNIDRVRKK----------------------------

FRPR-18a S-------RKPILNAHLSEPLIGRSSQ------TNLHAEKKRTLLVGVQR----------

FRPR-18c SS-----YRKPILNAHLSEPLIGRSSQ------TNLHAEKKRTLLVGVQR----------

FRPR-18b SSRSSDVLLKPIYMQKRSERFSSEYNERTCKHLAPFEEHKLPKLPSEKRK----------

EGL-6a S-------VSFVLYCTMSNEYLSTFRA-------LFFPWTRKNSLRGTRR----------

EGL-6b S-------VSFVLYCTMSNEYLSTFRA-------LFFPWTRKNSLRGTRR----------

NPR-3 GTDEARLIKKKPQSTSKMVSYPTNFSEIR-------------------------------

NPR-1 DRRKSDRIINQTSVYKTKIVHDTKHLNGRAKIGGGGSHEALKERELNSCSENLSYHVNG-

NPR-11 RVGLRSLRVVIFGEQESLKKSMRTEFRSRGGCKT--------------------------

**Supplementary Figure 3-5**

NPR-22a -----------------------------------------------------SSSETTL

NPR-22b -----------------------------------------------------QTCRSAR

GPR147 ----------------------------------------------------GRVAHHGL

GPR74 -----------------------------------------------------KSAEK--

NPR-10a ------------------------------------------------------SVTTTI

NPR-10b ------------------------------------------------------SVTTTI

NPR-4a GKVQPEAPSFKFNGSGRKKSAMMRILVQKRNAEEEEQLITKESPSPPEIQMDTLCAASII

NPR-4b GKVQPEAPSFKFNGSGRKKSAMMRILVQKRNAEEEEQLITKESPSPPEIQMDTLCAASII

NPR-4c -------------------------------------------PSK---------QHTLI

NPR-4d -------------------------------------------PSK---------QHTLI

NPR-5a ------------------------------------------------------CAGNSF

NPR-5b -------------------------------------------------QVVLDCEGSSY

FRPR-18a -----------------------------------------------------TNLQTPC

FRPR-18c -----------------------------------------------------TNLQTPC

FRPR-18b -----------------------------------------------------KKLHKMS

EGL-6a -----------------------------------------------------SWNHRHD

EGL-6b -----------------------------------------------------SWNHRHD

NPR-3 -------------------------------------------------------KETEI

NPR-1 ---------------------------------------------------HTRTPTPEV

NPR-11 -----------------------------------------------------VTTAEPA

NPR-22a PILSRSFSRIIKKIDLPCT-----------

NPR-22b FFEARPLVVVRNNSANSLA-----------

GPR147 PREGPGCSHLPLTIPAWDI-----------

GPR74 PQQELVMEELKETTNSSEI-----------

NPR-10a P-----------SSI---------------

NPR-10b P-----------SSI---------------

NPR-4a PRRKSAQPRSTNEKVVLPRKAS--------

NPR-4b PRRKSAQPRSTNEKVVLPRKASF-------

NPR-4c P----AMDNHTCQQV---------------

NPR-4d P----AMDNHTCQQV---------------

NPR-5a ------------------------------

NPR-5b TTSSRPLLIRTDVQATLSNGSTSTTREML-

FRPR-18a SFRRAQITKIAEREEEEETT----------

FRPR-18c SFRRAQITKIAEREEEEETT----------

FRPR-18b AVEHRGMPEITITFSEDLPDGEPDSPCQPC

EGL-6a DETKSPRTFLINRTAPSSYVGS--------

EGL-6b DETKSPRTFLINRTAPSSYVGS--------

NPR-3 ASTKTKITIAENDYRAGDQLL---------

NPR-1 QLNEVSSPEISKLVAEPEELIEFSVNDTLV

NPR-11 TFQRMNESMILSAMEQDEQL----------
